# Sense of belonging and academic outcomes among postsecondary students: a meta-analysis

**DOI:** 10.3389/fpsyg.2026.1841185

**Published:** 2026-08-05

**Authors:** Xuezhen Yang, Dekai Zhang, Xiaolin Wang, Shamsulariffin Bin Samsudin

**Affiliations:** 1 Faculty of Educational Studies, University Putra Malaysia, Serdang, Malaysia; 2 Daliuhang Primary School, Yantai, China; 3 Department of Physical Education, Ludong University, Yantai, China

**Keywords:** academic achievement, engagement, motivation, school belonging, self-efficacy

## Abstract

**Introduction:**

Sense of belonging is widely regarded as an important contributor to student success, yet its associations with academic outcomes in postsecondary education remain unclear.

**Methods:**

This meta-analysis synthesized evidence from 83 correlational studies published in peer-reviewed journals up to October 5, 2025, identified through Web of Science, ERIC, and Academic Search Complete. Random-effects models were used to estimate the associations between students’ sense of belonging and multiple academic outcomes. Subgroup analyses were conducted to examine variation across student characteristics, program duration, and measurement tools.

**Results:**

The results showed moderate positive correlations between school belonging and academic motivation, academic interest, and academic self-efficacy (*r* = 0.30, 0.44, and 0.36, respectively). Small positive correlations were found with academic performance, student retention, academic engagement, and persistence intention (*r* = 0.15, 0.18, 0.27, and 0.29, respectively). A small negative correlation was observed between school belonging and dropout intention (*r* = −0.24). Subgroup analyses showed stronger associations in STEM fields, classroom/course belonging, and longer academic programs.

**Discussion:**

The findings suggest that sense of belonging functions primarily as a motivational and psychosocial resource in postsecondary education, with stronger links to students’ motivation, confidence, and engagement than to direct indicators of academic performance. These results provide evidence for the importance of fostering belonging to support student success and retention in higher education.

**Systematic review registration:**

https://www.crd.york.ac.uk/PROSPERO/view/CRD42025638811, identifier CRD42025638811.

## Introduction

Sense of belonging has been consistently conceptualized as a fundamental psychological need and a central component of students’ academic development (Allen et al., 2021; Strayhorn, 2018). Within educational contexts, belonging reflects the extent to which students feel accepted, valued, and supported by peers, faculty, and the broader institutional community (Goodenow and Grady, 1993). A substantial body of research has linked students’ sense of belonging to positive academic and psychosocial outcomes, and prior evidence from K–12 settings suggests that stronger belonging is associated with better academic achievement, persistence, and adjustment (Akar Vural et al., 2020; Arslan, 2021; Korpershoek et al., 2020). These findings underscore the importance of belonging as a meaningful correlate of student success.

However, whether these conclusions can be generalized to postsecondary education remains unclear. Higher education differs from compulsory schooling in several important ways that may shape both the development and the consequences of belonging. Postsecondary students are often navigating the transition from adolescence to early adulthood while adapting to unfamiliar academic and social environments, making belonging both salient and potentially fragile (Strayhorn, 2018). In addition, belonging in higher education is often formed across multiple contexts, such as academic departments, classrooms, peer networks, and student organizations, rather than within a single stable school setting. Because enrollment is voluntary and institutional structures are often more fragmented, a weak sense of belonging may have more direct implications for disengagement, withdrawal, and dropout than in K–12 education (Lewis and Mathuews, 2020). These features suggest that the role of belonging in postsecondary education warrants separate examination.

The potential importance of belonging in postsecondary education is also supported by theory. From the perspective of Self-Determination Theory, belonging reflects the fulfillment of relatedness needs, which facilitates intrinsic motivation and the internalization of academic values (Ryan and Deci, 2024). From a Social Cognitive Theory perspective, belonging may also strengthen academic self-efficacy by fostering supportive social experiences and reinforcing students’ confidence in their academic capabilities (Bandura, 1986; Zumbrunn et al., 2014). Through these processes, belonging may contribute to greater engagement, persistence, and academic adjustment. Consistent with these perspectives, empirical studies in postsecondary settings have examined belonging in relation to a wide range of academic outcomes, including grade point average, retention, persistence, motivation, engagement, and self-efficacy. Although many studies report positive associations (Chen et al., 2021; Cwik and Singh, 2022; Li and Singh, 2023), the magnitude of these relationships varies considerably across the literature (Au et al., 2023; Hilts et al., 2018). Moreover, such variation may reflect differences in institutional settings, student populations, cultural contexts, and measurement approaches (Gopalan et al., 2022; Luciano-Wong and Crowe, 2019; Totonchi et al., 2023). As a result, it remains difficult to determine whether belonging shows a similar pattern of association across different academic outcome domains, or whether its links are stronger for some aspects of academic functioning than for others.

Taken together, the existing literature suggests that belonging is relevant to student success in higher education, but the overall pattern of associations remains difficult to determine from individual studies alone. Existing research has examined diverse academic outcomes and has used varied measures of belonging across different educational contexts, making it challenging to draw clear conclusions about the strength, consistency, and boundary conditions of these relationships. A comprehensive quantitative synthesis is therefore needed to clarify the associations between belonging and academic outcomes in postsecondary education and to examine whether these associations vary across outcome domains and study characteristics.

Therefore, this study aims to conduct a meta-analysis clarifying the relationship between academic belonging and postsecondary students’ academic outcomes. Specifically, it addresses two research questions: (1) What is the overall strength and direction of the association between belonging and academic outcomes (academic motivation, self-efficacy, engagement, persistence, and performance)? (2) How do individual characteristics (e.g., gender, region) and educational contexts (e.g., institution type) moderate these relationships? This study provides a comprehensive and nuanced understanding of how academic belonging contributes to student success across diverse postsecondary settings.

## Methods

### Literature search and inclusion criteria

This meta-analysis was conducted in accordance with the PRISMA guidelines (Page et al., 2021) (Prospero registration number: CRD42025638811). Articles published by October 5, 2025, were retrieved from Web of Science, ERIC, and PsycINFO using Boolean operators with various keywords to identify relevant research on the topic (see Supplementary Material 1 for search terms). Descendancy searches were conducted on the two most cited articles related to college belonging in the literature, Walton and Cohen (2007) and Goodenow (1993), each with over 3,000 citations, to identify additional studies potentially meeting the inclusion criteria. The search process involved an initial screening of titles and abstracts to exclude irrelevant studies, followed by the retrieval and evaluation of full-text documents for the remaining records. Two researchers conducted the search and screening independently, with a third researcher resolving any discrepancies to ensure accuracy and consistency.

The selection criteria were as follows: (1) The study was conducted in a postsecondary education context (e.g., community colleges or 4-year colleges). (2) The study examined the relationship between students’ sense of belonging and academic outcomes. Sense of belonging was defined as students’ perceived acceptance, inclusion, support, and connectedness within postsecondary educational contexts, including their connection to the institution, classroom or course, academic discipline, or peer community. Academic outcomes were categorized into performance outcomes and academic-related psychological indicators. Performance outcomes included objective indicators such as grades, GPA, test scores, retention, and dropout intention. Academic-related psychological indicators encompassed motivation and interest (e.g., academic motivation, academic interest), beliefs and persistence (e.g., academic self-efficacy, academic persistence), and behavior and engagement (e.g., academic engagement). (3) The study employed quantitative methods with a minimum sample size of 30 participants to ensure the statistical reliability and robustness of the findings.

### Data extraction

Data were systematically extracted from each study using a predefined Excel template to ensure consistency and accuracy. Data extraction and coding were performed independently by two researchers using a predefined template. Any discrepancies were resolved by a third researcher. The extracted information covered several key aspects: (1) study details, including the first author’s name, year of publication, and the country or region where the study was conducted; (2) sample characteristics, such as the total sample size and percentage of female participants; (3) sense of belonging measures, detailing the instruments used to assess this construct; (4) academic outcomes included motivation and interest (e.g., academic motivation and interest), beliefs and persistence (e.g., self-efficacy and persistence), behavior and engagement (e.g., academic engagement), and achievement and completion (e.g., academic performance, retention, and dropout intention); and (5) statistical associations, including correlation coefficients (r) and regression coefficients (β) that quantified the relationship between students’ sense of belonging and academic outcomes. The specific study characteristics are summarized in Table 1.

**TABLE 1:** Descriptive statistics of the included studies.

Authors	Country	N; % Female	Institution	School belonging construct	Outcomes	*r*
Aker and Şahin, 2022	Turkey	601, 55.2%	6-yr Medicine	Psychological Sense of School Membership Scale (Goodenow, 1993)	Academic engagement	r = 0.37
Alkan, 2016	Turkey	509, 51.1%	4-yr	Psychological Sense of School Membership Scale (Goodenow, 1993)	Drop out	r = −0.37
Au et al., 2023	Australia	141, 77.3%	4-yr public	Sense of Belonging Scale (Anderson-Butcher and Conroy, 2002)	Resilience	r = −0.41
					Academic performance	r = −0.16
Banchefsky et al., 2019	USA	516, 23%	4-yr public	Sense of Belonging Scale (Walton and Cohen, 2007)	Intent to persist	r = 0.23
					Academic performance	r = 0.55
					Self-efficacy	r = 0.51
Beatson et al., 2024	New Zealand	132, 55.3%	4-yr public	Sense of Belonging Scale (Yorke, 2016)	Motivation	r = 0.21
					Self-efficacy	r = 0.30
Brown et al., 2024	USA	638, 62.9%	4-yr public	Sense of Belonging Scale (Yeager et al., 2016)	Retention	r = 0.27
Buskirk-Cohen and Plants, 2019	USA	44, 71.1%	4-yr public	Revised Psychological Sense of School Membership Scale (Goodenow, 1993)	GPA	r = 0.19
					Academic commitment	r = 0.13
Carey et al., 2022	USA	416, 71.4%	4-yr	Sense of Belonging Scale (Walton and Cohen, 2007)	GPA	r = 0.19
Chen et al., 2021	USA	572, 75%	4-yr public	Sense of Belonging Scale (Walton and Cohen, 2007)	Class grade	r = 0.22
Chun et al., 2016	USA	289, 70.9%	4-yr public	Sense of Belonging Scale (Bollen and Hoyle, 1990)	Academic self-efficacy	r = 0.30
					GPA	r = −0.10
Cwik and Singh, 2021	USA	854, 62%	4-yr public	Psychological Sense of School Membership Scale (Goodenow, 1993)	Physics grade	r = 0.26
					Self-efficacy in physics	r = 0.53
					Interest in physics	r = 0.38
Cwik and Singh, 2022	USA	814, 64%	4-yr public	Psychological Sense of School Membership Scale (Goodenow, 1993)	Physics grade	r = 0.33
Davis et al., 2019	USA	837, 58%	4-yr public	Sense of Belonging Scale (Davis et al., 2019)	GPA	r = 0.06
Dueñas and Gloria, 2020	USA	236, 72%	4-yr public	Revised Psychological Sense of School Membership Scale (Goodenow, 1993)	Motivation	r = 0.03
Feser et al., 2024	Germany	263, 37.3%	3-yr public	Revised Sense of Belonging Scale (Good et al., 2012)	Intent to drop out	r = −0.46
Fink et al., 2020	USA	739, NR	4-yr private	Sense of Belonging Scale (Walton and Cohen, 2007)	Chemistry score	r = 0.31
Fong et al., 2023	USA	1393, 65%	2-yr	“Within a class or through another experience at this college, I have felt a sense of belonging.”	GPA	r = 0.14
Fong et al., 2025	USA	2403, 47.2%	2-yr public	“I feel welcome and respected at this college,” and “I have good relationships with others at this college.”	GPA	r = 0.15
Freeman et al., 2007	USA	238, 68.1%	4-yr public	Revised Psychological Sense of School Membership Scale (Goodenow, 1993)	GPA	r = 0.10
					Self-efficacy	r = 0.16
					Intrinsic motivation	r = 0.10
Gan et al., 2024	China	4463, 51.5%	4-yr public	University Identification Scale (Mael and Ashforth, 1992)	Student engagement	r = 0.32
					Academic achievement	r = 0.33
Gaudier-Diaz et al., 2019	USA	756, 75.3%	4-yr public	Social Connectedness Scale and Social Assurance Scale (Lee and Robbins, 1995)	GPA	r = −0.02
Gopalan and Brady, 2020	USA	23750, NR	2 and 4-yr	“I feel that I am a part of school.”	Persistence	r = 0.07
					First-year GPA	r = 0.10
Uslu Gülşen, 2024	Turkey	677, 68.4%	4-yr public	Sense of Belonging Scale (Hoffman et al., 2002)	Student engagement	r = 0.58
Hafer et al., 2021	USA	937, 55.7%	2-yr public	School Relatedness Scale (Furrer and Skinner, 2003)	GPA	r = −0.02
					Retention	r = 0.11
Han et al., 2017	USA	1400, 53.4%	4-yr public	Sense of Belonging subscale (Johnson et al., 2007)	Academic motivation	r = 0.32
					GPA	r = 0.26
					Retention	r = 0.09
Han et al., 2022	South Korea	2279, 53%	4-yr public	Sense of Belonging scale (Johnson et al., 2007)	Self-efficacy	r = 0.24
					Academic motivation	r = 0.31
					Academic engagement	r = 0.27
					GPA	r = 0.07
Hartley, 2011	USA	605, 70.6%	4-yr	Perceived Cohesion Scale (Bollen and Hoyle, 1990)	GPA	r = −0.06
Hausmann et al., 2009	USA	1365, 59.5%	4-yr public	Sense of Belonging Scale (Bollen and Hoyle, 1990)	Intent to persist	r = 0.23
Hilts et al., 2018	USA	206, 73.3%	4-yr public	Feelings of Relatedness Scale (Robnett, 2013)	Intention to leave	r = −0.15
Jackson et al., 2023	USA	4708, 57.1%	4-yr public	Sense of Belonging Scale (Jackson et al., 2023)	Intent to persist	r = 0.36
Kahalon et al., 2023	Israel	90, 91.1%	3-yr public	Social and Academic Fit Scale (Walton and Cohen, 2007)	GPA	r = 0.14
Kelly et al., 2024	Australia	1190, 69.5%	4-yr public	Sense of Belonging Index (Organisation for Economic Co-operation and Development [OECD], 2004)	Enjoyment	r = 0.41
					Motivation	r = 0.36
Kennedy and Tuckman, 2013	USA	2044, 60.4%	4-yr public	Psychological Sense of School Membership Scale (Goodenow, 1993)	GPA	r = 0.05
					Self-efficacy	r = 0.55
Knekta et al., 2020	Sweden	938, NR	4-yr public	Psychological Sense of School Membership Scale (Goodenow, 1993)	Engagement	r = 0.46
Krause-Levy et al., 2021	USA	5335, 31.8%	4-yr public	Sense of Belonging Scale (Anderson-Butcher and Conroy, 2002)	Retention	r = 0.26
Lewis and Hodges, 2015	USA	1089, 65.7%	4-yr public	Sense of Belonging Scale (Walton and Cohen, 2007)	GPA	r = 0.14
					Intent to persist	r = 0.51
					Domain motivation	r = 0.59
Lewis et al., 2017	USA	2590, 29.7%	4-yr	Sense of Belonging Scale (Walton and Cohen, 2007)	Self-efficacy	r = 0.38
					Intent to leave	r = −0.39
Li and Singh, 2023	USA	533, 39.6%	4-yr	Sense of Belonging Scale (Walton and Cohen, 2007)	Grade	r = 0.39
Liang et al., 2023	China	1045, 44.5%	4-yr public	Psychological Sense of School Membership Scale (Goodenow, 1993)	Student engagement	r = 0.33
Luo and Zhou, 2024	China	246, 65%	4-yr public	Chinese Version Psychological Sense of School Membership Scale (Goodenow, 1993)	Academic engagement	r = 0.30
					GPA	r = 0.16
Lytle and Shin, 2020	USA	1201, 30.9%	4-yr	Sense of Belonging Scale (Good et al., 2012)	STEM interest	r = 0.66
					STEM efficacy	r = 0.63
Maluenda-Albornoz et al., 2022	Chile	700, 59.7%	4-yr public	Membership factor of the Organizational Identification Questionnaire (Yáñez et al., 2006)	Student engagement	r = 0.48
Marksteiner et al., 2019	Germany	106, 69.8%	3-yr	Sense of Belonging Index (PISA; Organisation for Economic Co-operation and Development [OECD], 2013)	Grade	r = −0.11
					Intent to persist	r = 0.20
Matheka et al., 2024	Netherlands	476, 35.9%	3-yr	Sense of Belonging Scale (Meeuwisse et al., 2010)	Self-efficacy	r = 0.34
McClain et al., 2021	USA	400, 44.5%	4-yr public	Sense of Belonging Scale (Hagerty and Patusky, 1995).	Academic motivation	r = 0.20
					College self-efficacy	r = 0.53
Mcdermott et al., 2020	USA	933, 89.4%	4-yr	Sense of Belonging Scale (Walton and Cohen, 2007)	Resilience	r = 0.26
					Academic persistence	r = 0.21
McGee and Kruger, 2022	USA	106, 78.3%	4-yr public	Sense of Belonging Scale (Parada, 2019)	Academic engagement	r = −0.08
Menkor et al., 2021	Germany	719, 56%	3-yr	Psychological Sense of School Membership Scale (Goodenow, 1993)	Academic motivation	r = 0.20
					Dropout intention	r = −0.19
Mtshweni, 2024a	South Africa	489, 71.2%	University	Psychological Sense of School Membership Scale (Goodenow, 1993)	Intrinsic motivation	r = 0.19
					Academic persistence	r = 0.33
Mtshweni, 2024b	South Africa	955, 71.7%	University	Psychological Sense of School Membership Scale (Goodenow, 1993)	Academic persistence	r = 0.43
Okolie et al., 2021	Nigeria	509, 27.3%	4-yr public	Sense of Belonging Scale (Anderson-Butcher and Conroy, 2002).	Self-efficacy	r = −0.07
					Student engagement	r = 0.02
Pedler et al., 2022	Australia	578, 70.8%	University	Sense of Belonging Index (PISA; Organisation for Economic Co-operation and Development [OECD], 2013)	Enjoyment	r = 0.41
					Motivation	r = 0.34
Pittman and Richmond, 2007	USA	266, 51%	4-yr	Psychological Sense of School Membership Scale (Goodenow, 1993)	University grades	r = 0.21
Rattan et al., 2018	UK	69, 96.7%	4-yr	Sense of Belonging Scale (Cheryan and Plaut, 2010)	Final course grade	r = 0.43
Reid et al., 2020	UK	3310, 66.3%	University	Sense of Belonging Scale (Walton and Cohen, 2007)	Academic performance	r = 0.21
Reid et al., 2020	UK	384, 74.4%	University	Sense of Belonging Scale (Walton and Cohen, 2007)	Academic performance	r = 0.15
Saroughi and Kitsantas, 2021	USA	502, 56.2%	4-yr public	Psychological Sense of School Membership Scale (Goodenow, 1993)	Self-efficacy	r = 0.49
Schar et al., 2017	USA	83, 52%	4-yr private	Sense of Belonging Scale (Walton and Cohen, 2007)	Self-Efficacy	r = 0.59
					Grade Performance	r = 0.23
Seyranian et al., 2018	USA	160, 27.5%	4-yr public	Sense of Belonging Scale (Seyranian et al., 2018)	Physics grade	r = 0.10
Shook and Clay, 2012	USA	159, 81%	4-yr	“I feel a sense of belonging to the university.” “How much do you identify with your university?”	GPA	r = 0.03
Smith et al., 2013	USA	149, 50.3%	4-yr public	Sense of Belonging Scale (Walton and Cohen, 2007)	Domain motivation	r = 0.65
					GPA	r = 0.43
Sotardi et al., 2022	New Zealand	896, 55.4%	4-yr public	Sense of Belonging Scale (Hoffman et al., 2002)	Academic self-efficacy	r = 0.40
					Class enjoyment	r = 0.36
					GPA	r = 0.04
Strayhorn, 2020	USA	247, 58%	4-yr	Sense of Belonging Scale (Hagerty et al., 1992)	Intent to persist	r = 0.41
Suhlmann et al., 2018	Germany	367, 74%	4-yr	Psychological Sense of School Membership Scale (Goodenow, 1993)	Academic motivation	r = 0.41
					Dropout intention	r = −0.20
Swanson et al., 2021	USA	1702, NR	4-yr public	Sense of Belonging Scale (Swanson et al., 2021)	Academic self-efficacy	r = 0.51
					Cumulative GPA	r = 0.18
Takimoto et al., 2021	USA	153, 56.2%	4-yr	Sense of Belonging Scale (Good et al., 2012)	Academic self-efficacy	r = 0.40
Tao et al., 2008	China	164, 75.6%	4-yr	Psychological Sense of School Membership Scale (Goodenow, 1993)	GPA	r = 0.23
Thomas et al., 2014	USA	139, NR	4-yr public	University Environment Scale (Gloria and Robinson Kurpius, 1996)	Campus involvement	r = 0.07
					Self-efficacy	r = 0.38
					Self-reported persistence	r = 0.23
Townley et al., 2013	USA	53, 42%	4-yr public	Sense of Community Index (Perkins et al., 1990).	GPA	r = 0.05
van Herpen et al., 2020	Netherlands	237, 82.3%	3-yr public	Sense of Belonging Scale (Meeuwisse et al., 2010)	GPA	r = −0.02
					Retention	r = 0.06
van Lamoen et al., 2024	Netherlands	2070, 68.3%	3-yr public	Sense of Belonging Scale (Meeuwisse et al., 2010)	GPA	r = 0.02
					Academic self-efficacy	r = 0.24
Vayre and Vonthron, 2019	France	506, 71.7%	3-yr public	Sense of Belonging Scale (Rovai, 2002)	Academic self-efficacy	r = 0.03
					Perseverance	r = 0.25
					Passing exams	r = 0.01
Vázquez et al., 2024	Spain	2261, 80.6%	4-yr	Sense of Belonging Scale (Yeager et al., 2016)	Course grade	r = 0.19
Veldman et al., 2023	USA	829, 88%	3-yr public	Need to Belong Scale (Leary et al., 2013)	Academic engagement	r = −0.28
Verdín, 2021	USA	373, 100%	4-yr	Sense of Belonging Scale (Verdín and Godwin, 2018)	Engineering interest	r = 0.56
Wells and Horn, 2015	USA	116, 78.4%	4-yr public	Cultural Congruity Scale and University Environment Scale (Gloria and Robinson Kurpius, 1996)	GPA	r = −0.15
Wilcox and Nordstokke, 2019	Canada	71, 73.2%	4-yr public	College Student Subjective Well-being Questionnaire (Renshaw, 2018)	Academic efficacy	r = 0.60
Wilson et al., 2018	USA	611, 75.6%	4-yr public	Psychological Sense of School Membership Scale (Goodenow, 1993)	GPA	r = 0.16
Wilson et al., 2020	USA	470, 79%	4-yr public	Psychological Sense of School Membership Scale (Goodenow, 1993)	GPA	r = 0.20
Witherspoon and Schunn, 2022	USA	416, 58%	4-yr public	Sense of Belonging Scale (Witherspoon and Schunn, 2022)	Physics Retention	r = 0.24
					Physics GPA	r = 0.38
Wolf et al., 2021	Germany	925, 68.2%	4-yr public	Sense of Belonging Index (PISA; Organisation for Economic Co-operation and Development [OECD], 2013)	Dropout intention	r = −0.10
					GPA	r = 0.02
Yust et al., 2021	USA	508, 56.3%	4-yr private	Sense of Belonging Scale (Asher and Weeks, 2014)	Final course grade	r = 0.25
					Involvement in research	r = 0.30
					Course engagement	r = 0.62
Zumbrunn et al., 2014	USA	212, 73%	4-yr public	Psychological Sense of School Membership Scale (Goodenow, 1993)	Self-efficacy	r = 0.44
					Engagement	r = 0.11
					Achievement	r = 0.06

### Quality assessment of studies

The methodological quality of the included studies was assessed using the Newcastle-Ottawa Scale (NOS) for cross-sectional studies (Stang, 2010). The NOS evaluates studies across three domains: selection, comparability, and outcome assessment, with a maximum score of 9. Studies scoring 7 or higher were classified as high quality, those scoring 4–6 as moderate quality, and those scoring below 4 as low quality. Two reviewers independently assessed the methodological quality of each included study. Any discrepancies were resolved through discussion, and when consensus could not be reached, a third reviewer was consulted.

### Data analyses

The meta-analysis was performed using R software. The metagen function from the meta package was employed to conduct the meta-analysis, while the funnel function was used to generate funnel plots for assessing publication bias. Egger’s test for bias detection was carried out using the metabias function from the metafor package. Subgroup analyses were performed using the rma function in metafor. Correlation coefficients (r) were used as the primary effect size. Fisher’s Z-transformed correlation coefficients were utilized during the synthesis process to stabilize variance and enhance the accuracy of pooled estimates (Lei et al., 2018). The Fisher Z transformation is given by:


$$Z=\frac{1}{2}\,\ln\left(\frac{1+r}{1-r}\right)$$

where Z is the Fisher Z-transformed value and r is the correlation coefficient. After meta-analysis, the Z values were converted back to correlation coefficients for intuitive interpretation. To account for variability across studies, a random-effects model was employed, recognizing potential heterogeneity due to differences in study populations, methodologies, or contexts. The magnitude of correlation coefficients was categorized as small (0.1 ≤ r < 0.3), medium (0.3 ≤ r < 0.5), and large (r ≥ 0.5) (Cohen, 2013). Statistical significance was defined at *p* < 0.05.

Subgroup analyses were performed based on region, academic discipline, gender, institution type, publication year, belonging type, and the method of measuring sense of belonging. Regions were categorized into Asia-Pacific, Europe, and the Americas. Academic discipline was classified into STEM fields, health sciences, business, social sciences, and mixed disciplines. Given that most studies included mixed-gender samples, the effect of the percentage of female participants on the outcomes was examined. The female percentage was divided into three groups: low ( < 50%), medium (50−75%), and high ( > 75%). Institution type was classified as 4-year versus less than 4-year programs. Belonging type was classified into institutional belonging, classroom or course belonging, disciplinary belonging, social belonging, and mixed belonging. Publication year was grouped based on sample distribution as follows: before 2015, 2016–2020, and 2021 or later. School belonging scales were the Sense of Belonging Scale (Walton and Cohen, 2007) and the Psychological Sense of School Membership Scale (Goodenow, 1993). Accordingly, the measurement methods were classified into three groups: Walton’s scale, Goodenow’s scale, and other scales.

## Results

### Study selection and study characteristics

**FIGURE 1:**
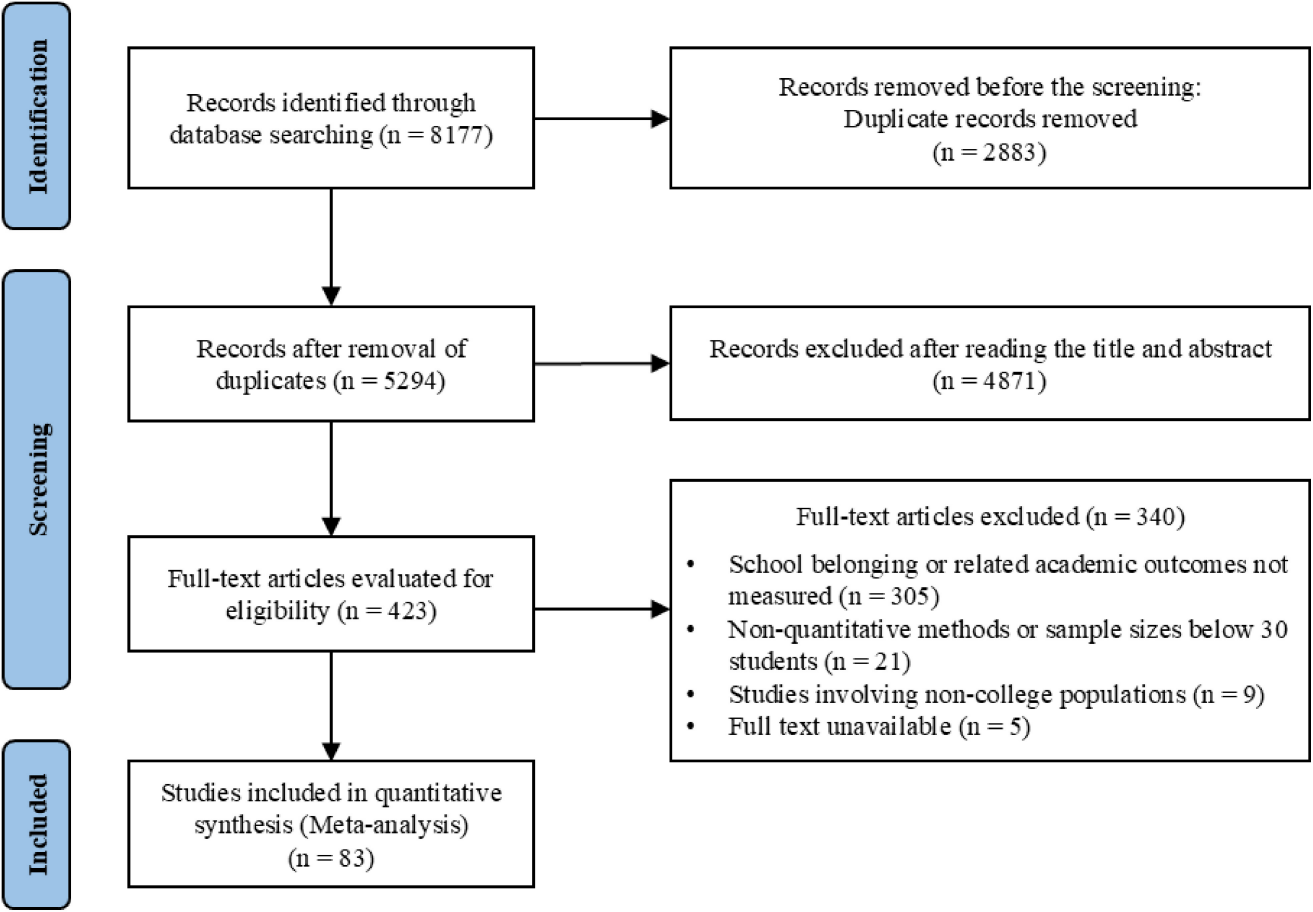
PRISMA flow diagram.

Figure 1 presents the study selection process. The initial search identified 8,177 records, of which 2,883 duplicates were removed. After title and abstract screening, 423 full-text articles were assessed for eligibility. Of these, 340 were excluded because they did not measure school belonging or relevant academic outcomes (*n* = 305), used non-quantitative methods or had sample sizes below 30 students (*n* = 21), involved non-college populations (*n* = 9), or had unavailable full texts (*n* = 5). Finally, 83 studies were included in the quantitative synthesis.

The 83 included studies involved 93,461 students, with sample sizes ranging from 44 to 23,750. Most studies were conducted in 4-year higher education institutions, although some involved 2-year, 3-year, or broader university contexts. The studies covered diverse countries, with a large proportion from the United States. School belonging was measured using various instruments, most commonly Goodenow’s Psychological Sense of School Membership Scale and Walton and Cohen’s sense of belonging measure. The outcomes included academic performance, motivation, interest, self-efficacy, engagement, persistence intention, retention, and dropout intention.

### Quality assessment of studies

The NOS scores ranged from 4 to 8, with a median score of 6. Among the 83 included studies, 41 were classified as high quality and 42 as moderate quality, and no study was rated as low quality. Overall, the included studies showed moderate to high methodological quality, indicating that the evidence base was generally suitable for quantitative synthesis. Detailed NOS ratings are provided in Supplementary Material 2.

### Meta-analysis results

Table 2 presents the pooled correlations between school belonging and the four academic outcome domains. Overall, school belonging showed small to moderate positive correlations with all academic outcomes. Specifically, moderate correlations were observed between school belonging and academic motivation (*r* = 0.30), academic interest (*r* = 0.44), and academic self-efficacy (*r* = 0.36). Small correlations were found with academic performance (*r* = 0.15), student retention (*r* = 0.18), academic engagement (*r* = 0.27), and persistence intention (*r* = 0.29). A negative association was found with dropout intention (*r* = −0.24). Forest plots for these meta-analyses are presented in Supplementary Material 3. Funnel plots for meta-analyses displayed generally symmetrical distribution, suggesting no significant publication bias (see Supplementary Material 4 for funnel plots). Egger’s test indicated no significant publication bias (t = −0.68 to 0.784, *p* > 0.05). The I^2^ statistic was high, ranging from 89.2 to 98.5%, indicating significant heterogeneity, which prompted the use of subgroup analyses to explore potential sources of variation. Sensitivity analyses supported the robustness of the findings, with the leave-one-out procedure indicating that no single study exerted an undue influence on the pooled estimates (see Supplementary Material 5).

**TABLE 2:** The relationship between sense of belonging and academic outcomes.

Academic outcomes	Variables	*k*	ES (95% CI)	*p*	*I*^2^ (%)	RW (%)
Motivation and interest	AM	13	0.30 (0.21, 0.39)	***	94.8	7.0−8.1
	AI	6	0.44 (0.35, 0.51)	***	96.3	15.9–17.0
Beliefs and persistence	AS	20	0.36 (0.29, 0.42)	***	96.4	4.0–5.3
	PER	12	0.29 (0.22, 0.35)	***	98.5	6.1–9.3
Behavior and engagement	AE	14	0.27 (0.13, 0.39)	***	97.9	6.6–7.4
Achievement and completion	AP	48	0.15 (0.11, 0.19)	***	93.9	1.2–2.4
	SR	7	0.18 (0.12, 0.23)	***	89.2	10.3–16.9
	DI	6	−0.24 (−0.34, −0.13)	***	89.6	15.1–17.8

AM, academic motivation; AI, academic interest; AS, academic self-Efficacy; AE, academic engagement; AP, academic performance; SR, student retention; DI, dropout intention; PER, academic persistence. ***p < 0.001.

Table 3 presents the results of the subgroup analyses. The findings indicate that neither region nor the percentage of female participants moderated the relationship between school belonging and academic outcomes. However, four factors did show moderating effects: academic discipline, belonging type, academic program duration, and the type of belonging measure. For academic discipline, the association between school belonging and academic outcomes was stronger in STEM fields than in social sciences and mixed disciplines. For the belonging type, classroom/course belonging showed a stronger association with academic outcomes than institutional and disciplinary belonging. For academic program duration, students enrolled in 4-year or longer programs showed a stronger association between school belonging and academic outcomes than those in shorter programs. For the type of school belonging measure, the association was strongest when Walton’s scale was used, followed by Goodenow’s scale, with the weakest effect observed for other measures.

**TABLE 3:** Subgroup analysis.

Outcome	Moderator variable	Category	k	ES	SE	*z*-value	*p*-value	95% CI	Q_M_	*p*-value
GPA	Female %	< 50%	5	0.280	0.108	2.593	**	[0.068, 0.492]	3.555	0.059
		50%–75%	28	0.143	0.027	5.230	***	[0.090, 0.197]		
		> 75%	12	0.106	0.046	2.693	**	[0.015, 0.196]		
	Field of study	STEM	13	0.302	0.050	6.021	***	[0.204, 0.401]	17.179	***
		Social sciences	4	0.017	0.024	0.721	0.471	[−0.030, 0.064]		
		Mixed	8	0.043	0.053	0.812	0.417	[−0.061, 0.146]		
	Belonging type	Institutional	28	0.103	0.024	4.262	***	[0.055, 0.150]	12.636	**
		Classroom/course	7	0.309	0.090	3.439	***	[0.132, 0.485]		
		Disciplinary	11	0.218	0.040	5.504	***	[0.140, 0.296]		
	Region	Asia	5	0.129	0.085	1.511	0.131	[−0.038, 0.296]	1.533	0.465
		Europe	9	0.096	0.044	2.199	*	[0.011, 0.182]		
		Americas	34	0.171	0.028	6.062	***	[0.116, 0.226]		
	Measures	(Goodenow)	10	0.189	0.033	5.738	***	[0.125, 0.254]	15.568	**
		(Walton)	11	0.284	0.048	5.984	***	[0.191, 0.378]		
		(Others)	27	0.088	0.029	3.063	**	[0.032, 0.144]		
	Institution	< 4 years	8	0.049	0.031	1.575	0.115	[−0.012, 0.109]	4.911	*
		≥ 4 years	40	0.175	0.026	6.709	***	[0.124, 0.226]		
	Publish year	Before 2015	11	0.103	0.046	2.215	*	[0.012, 0.194]	1.311	0.252
		2016–2020	18	0.156	0.044	3.565	***	[0.070, 0.242]		
		2021–2025	19	0.177	0.033	5.310	***	[0.111, 0.242]		
Motivation	Female %	< 50%	11	0.355	0.063	5.651	***	[0.232, 0.478]	1.758	0.185
		50%–75%	2	0.158	0.048	3.274	**	[0.063, 0.252]		
	Field of study	STEM	4	0.538	0.058	9.340	***	[0.425, 0.650]	10.338	**
		Mixed	3	0.161	0.117	1.380	0.168	[−0.068, 0.389]		
	Region	Asia	4	0.339	0.020	17.320	***	[0.300, 0.377]	0.006	0.997
		Europe	2	0.317	0.117	2.719	**	[0.088, 0.545]		
		Americas	7	0.329	0.107	3.061	**	[0.118, 0.539]		
	Measures	(Goodenow)	4	0.218	0.081	2.681	**	[0.059, 0.377]	15.568	***
		(Walton)	2	0.696	0.037	18.597	***	[0.622, 0.769]		
		(Others)	7	0.285	0.036	8.026	***	[0.215, 0.355]		
	Publish year	Before 2015	3	0.517	0.210	2.466	*	[0.106, 0.929]	2.972	0.085
		2016–2020	3	0.270	0.119	2.274	*	[0.037, 0.503]		
		2021–2025	7	0.274	0.032	8.523	***	[0.211, 0.336]		
Self-efficacy	Female %	< 50%	5	0.366	0.118	3.114	**	[0.136, 0.597]	0.030	0.863
		50%–75%	13	0.385	0.053	7.247	***	[0.281, 0.489]		
	Belonging type	Institutional	11	0.406	0.042	9.771	***	[0.325, 0.487]	1.293	0.731
		Classroom/course	5	0.380	0.124	3.054	**	[0.136, 0.624]		
		Disciplinary	2	0.477	0.081	5.860	***	[0.317, 0.636]		
		Social	2	0.258	0.328	0.785	0.423	[−0.385, 0.900]		
	Region	Asia	2	0.399	0.046	8.692	***	[0.309, 0.489]	3.597	0.309
		Europe	3	0.210	0.093	2.264	*	[0.028, 0.392]		
		Americas	12	0.437	0.063	6.972	***	[0.314, 0.560]		
	Measures	(Goodenow)	4	0.576	0.027	21.679	***	[0.524, 0.628]	8.985	*
		(Walton)	3	0.522	0.078	6.715	***	[0.370, 0.675]		
		(Others)	13	0.306	0.054	5.685	***	[0.200, 0.411]		
	Publish year	Before 2015	4	0.418	0.099	4.232	***	[0.225, 0.612]	0.067	0.796
		2016–2020	5	0.388	0.109	3.553	***	[0.174, 0.602]		
		2021–2025	11	0.383	0.061	6.315	***	[0.264, 0.502]		
Engagement	Region	Asia	4	0.314	0.017	18.037	***	[0.280, 0.348]	3.834	0.147
		Europe	3	0.516	0.079	6.532	***	[0.361, 0.671]		
		Americas	7	0.157	0.135	1.166	0.244	[−0.107, 0.421]		
	Measures	(Goodenow)	5	0.338	0.061	5.578	***	[0.219, 0.457]	0.229	0.633
		(Others)	9	0.252	0.116	2.176	*	[0.025, 0.479]		
Persistence	Region	Europe	2	0.242	0.041	5.898	***	[0.161, 0.322]	0.520	0.471
		Americas	10	0.316	0.048	6.613	***	[0.222, 0.410]		
	Measures	(Goodenow)	2	0.406	0.058	6.955	***	[0.291, 0.520]	1.913	0.384
		(Walton)	3	0.338	0.114	2.963	**	[0.114, 0.561]		
		(Others)	7	0.256	0.049	5.221	***	[0.160, 0.352]		
	Publish year	Before 2015	3	0.349	0.113	3.101	**	[0.129, 0.570]	0.141	0.707
		2016–2020	6	0.227	0.050	4.567	***	[0.129, 0.324]		
		2021–2025	3	0.395	0.032	12.189	***	[0.331, 0.458]		
Interest	Region	Asia	3	0.416	0.021	20.097	***	[0.375, 0.456]	2.681	0.102
		Americas	3	0.609	0.116	5.266	***	[0.382, 0.836]		
Dropout	Measures	(Goodenow)	3	−0.261	0.064	−4.082	***	[−0.386, −0.136]	0.015	0.903
		(Others)	3	−0.246	0.123	−2.007	*	[−0.486, −0.006]		

Subgroup analyses required at least three sufficiently homogeneous studies per subgroup. *p < 0.05, **p < 0.01, ***p < 0.001.

## Discussion

The present meta-analysis synthesized the associations between sense of belonging and academic outcomes among postsecondary students. Overall, the findings indicate that belonging is positively associated with a broad range of academic outcomes, although the strength of these associations differs across outcome domains. Moderate correlations were observed for motivation, interest, self-efficacy, persistence, and engagement, whereas smaller correlations were found for academic performance and retention. In addition, belonging was negatively associated with dropout intention. Subgroup analyses further showed that these associations varied according to academic discipline, belonging type, academic program duration, with stronger associations generally observed in STEM fields, classroom/course belonging, and longer programs. By contrast, no significant moderating effects were identified for region or the percentage of female participants.

This meta-analysis identified a significant yet small positive association between sense of belonging and academic performance (e.g., GPA or test scores), consistent with the meta-analysis by Korpershoek et al. (2020) in secondary education. Expanding this scope, the current research supports the relevance of belonging within postsecondary education contexts. Furthermore, this meta-analysis revealed that students with a heightened sense of belonging were found to demonstrate greater academic persistence and lower dropout rates, reflecting the value of belonging as a key psychosocial resource. Belonging may be linked to emotional security and social connection, which are also associated with students’ ability to navigate academic challenges and remain engaged in their educational pursuits (Strayhorn, 2018). These findings underscore the importance of institutional efforts to cultivate a sense of belonging through inclusive campus climates, meaningful faculty-student interactions, and peer support networks. Such initiatives may be relevant to more favorable academic outcomes and retention, particularly in environments characterized by significant academic and social demands.

The meta-analysis further revealed that the associations between sense of belonging and motivation, interest, self-efficacy, persistence, and engagement were stronger than its association with academic performance. This pattern suggests that belonging may be more closely linked to academic functioning through affective and behavioral processes than through a direct association with performance outcomes such as GPA or test scores. Students who feel accepted and supported are more likely to sustain attention, invest effort, and persist when facing academic challenges (Mtshweni, 2024a). The stronger associations with motivation, engagement, and self-efficacy, together with the relatively small association with performance, suggest that belonging may be more closely connected to students’ daily academic experiences than to performance indicators alone. The relatively weak link with performance outcomes highlights the multifaceted nature of academic achievement, which is influenced by prior preparation, instructional quality, and assessment practices that may weaken the observed association between belonging and performance (Imran et al., 2023). This finding is consistent with earlier meta-analytic evidence in secondary education (Korpershoek et al., 2020) and extends it to postsecondary settings, suggesting that belonging should be regarded less as a direct determinant of grades and more as a foundational psychosocial resource associated with motivation, confidence, and persistence. From a practical standpoint, this distinction underscores that efforts to strengthen belonging may be more closely reflected in students’ daily engagement and resilience than in immediate improvements in measurable performance, reinforcing the need for institutions to foster inclusive climates, supportive faculty–student interactions, and strong peer networks.

Notably, the duration of academic programs significantly moderates the relationship between sense of belonging and academic performance. This study found that in longer academic programs, the association between belonging and academic outcomes is stronger. Extended programs may provide students with more time to immerse themselves in the campus environment, fostering stable social connections and meaningful faculty-student interactions. These sustained relationships and emotional support may be associated with students’ academic motivation, self-efficacy, and engagement, which may partly explain the stronger association with academic performance (Gopalan and Brady, 2020). Furthermore, students in longer programs may face greater academic demands and prolonged challenges, making them more reliant on the emotional security and social resources provided by a strong sense of belonging to sustain their persistence and resilience. In contrast, students in shorter programs, due to time constraints, may have fewer opportunities to establish deep social connections, potentially weakening the association between belonging and academic outcomes. Therefore, institutions offering shorter programs should prioritize strategies to rapidly cultivate a sense of belonging, such as orientation programs, structured peer collaborations, and faculty mentoring initiatives, to help students build meaningful connections within a limited timeframe and support positive academic experiences.

Academic discipline also moderated the association between sense of belonging and academic performance, with a stronger association observed in STEM fields than in social sciences and mixed disciplinary samples. This pattern may reflect the relatively structured and demanding nature of many STEM programs, where coursework, assessments, laboratory activities, and project-based tasks may make academic confidence, engagement, and persistence particularly relevant to students’ academic outcomes. In such contexts, a stronger sense of belonging may be more closely linked to students’ academic integration and sustained engagement. This interpretation is consistent with previous research highlighting the importance of STEM-specific belonging for students’ engagement, self-efficacy, persistence, and academic adjustment (Hansen et al., 2024; Rainey et al., 2018). By contrast, social sciences and mixed disciplinary samples may involve more diverse learning structures, assessment formats, and student experiences, which may dilute the observed association between belonging and academic performance.

In addition to program duration and academic discipline, the type of belonging assessed also moderated the association between sense of belonging and academic performance. Specifically, classroom/course belonging showed a stronger association with academic performance than institutional belonging and disciplinary belonging. This pattern may be because classroom- or course-level belonging is more closely connected to students’ immediate learning experiences, including teacher-student interaction, peer collaboration, course engagement, and perceived support within specific academic settings (Gillen-O’Neel, 2021; Verbree et al., 2025). These proximal learning experiences are more closely related to students’ daily academic participation and performance-related behaviors. By contrast, institutional belonging reflects students’ broader connection to the university, while disciplinary belonging captures identification with a field of study. Although these forms of belonging are important for students’ overall academic adjustment, they may be less closely aligned with short-term academic performance indicators. Differences in measurement tools may further contribute to this variation, as different scales emphasize different aspects of belonging, such as academic support, emotional connection, or social identification. Therefore, educational practices and student support strategies should pay greater attention to students’ belonging within specific courses and learning environments, rather than focusing only on general institutional attachment.

### Limitations

This meta-analysis has several limitations that warrant consideration. First, the predominance of cross-sectional designs precludes causal inferences; the observed associations may reflect bidirectional patterns, and psychological variables such as motivation and self-efficacy may mediate or moderate the belonging-outcome link—dynamics that require further investigation through longitudinal or experimental designs. Second, the effect sizes were generally small to moderate, suggesting that the direct association between belonging and academic outcomes may be relatively modest in magnitude. Third, measurement tool diversity poses validity concerns, as instruments emphasized different dimensions of belonging (e.g., academic vs. emotional belonging) and some lacked adequate validity information, limiting cross-study comparability and highlighting the need for a standardized measurement framework. Finally, although no significant regional moderating effect was detected, geographical imbalance remains an important limitation, as most included studies were conducted in the Americas, particularly the United States (60.9%). This uneven distribution may limit the generalizability of the findings to other cultural and educational contexts and may obscure potential cultural differences in how sense of belonging is experienced and associated with academic performance.

## Conclusion

This meta-analysis indicates that sense of belonging is positively associated with multiple academic outcomes among postsecondary students, but the strength of these associations differs across outcome domains. Belonging was more strongly related to motivation, interest, self-efficacy, persistence, and engagement than to academic performance and retention, and it was negatively associated with dropout intention. The association with academic performance appeared stronger in longer academic programs, STEM disciplines, and studies assessing classroom/course belonging. These findings highlight belonging as an important psychosocial resource for student success and support institutional efforts to cultivate inclusive and supportive academic environments. Future research should investigate contextual variation and use longitudinal or intervention designs to better clarify the direction and pathways of these associations.

## Data Availability

The original contributions presented in this study are included in this article/Supplementary material, further inquiries can be directed to the corresponding authors.
