# Supplementary material for: Sense of belonging and academic outcomes among postsecondary students: a meta-analysis
Source: Front Psychol. 2026 Aug 5;17:1841185. doi: 10.3389/fpsyg.2026.1841185 (PMC13485907; doi:10.3389/fpsyg.2026.1841185)
Supplement: Supplementary file 1 [file Data_Sheet_1.pdf]

Supplementary Material 1

Search Terms

Web of Science (4463)
("academic performance" OR "academic achievement" OR "GPA" OR "test scores" OR "exam scores" OR "academic grades" OR "academic persistence" OR "student persistence" OR "retention" OR "graduation rates" OR "degree completion" OR "academic motivation" OR "student motivation" OR "academic engagement" OR "student engagement" OR "academic interest" OR "dropout") AND ("school belonging" OR "sense of belonging" OR "school connectedness" OR "school attachment" OR "student belonging" OR "school membership" OR "identification with school" OR "commitment") AND (“college” OR “university”)

ERIC (3156)
(Journal Articles OR Meeting Papers) AND ("academic performance" OR "academic achievement" OR "GPA" OR "test scores" OR "exam scores" OR "academic grades" OR "academic persistence" OR "student persistence" OR "retention" OR "graduation rates" OR "degree completion" OR "academic motivation" OR "student motivation" OR "academic engagement" OR "student engagement" OR "academic interest" OR "dropout") AND ("school belonging" OR "sense of belonging" OR "school connectedness" OR "school attachment" OR "student belonging" OR "school membership" OR "identification with school" OR "commitment") AND (“college” OR “university”)

Academic Search Complete (558)
AB ("academic performance" OR "academic achievement" OR "GPA" OR "test scores" OR "exam scores" OR "academic grades" OR "academic persistence" OR "student persistence" OR "retention" OR "graduation rates" OR "degree completion" OR "academic motivation" OR "student motivation" OR "academic engagement" OR "student engagement" OR "academic interest" OR "dropout") AND AB ("school belonging" OR "sense of belonging" OR "school connectedness" OR "school attachment" OR "student belonging" OR "school membership" OR "identification with school" OR "commitment") AND AB (“college” OR “university”)

Supplementary Material 2

Table S1. Newcastle-Ottawa Scale (Adapted for Cross-Sectional Studies).

**Studies**	**Selection**				**Comparability**	**Outcome**		**Total NOS**
	**1**	**2**	**3**	**4**	**5**	**6**	**7**	
Aker & Şahin 2022	1	1	0	2	1	1	1	7
Alkan 2015	1	1	0	2	0	1	1	6
Au et al., 2023	1	0	0	2	1	1	1	6
Banchefsky et al., 2019	1	1	0	2	1	1	1	7
Beatson et al., 2024	1	1	0	2	1	1	1	7
Brown et al., 2024	1	1	0	2	0	1	1	6
Buskirk-Cohen & Plants 2019	1	0	1	2	0	1	1	6
Carey et al., 2022	1	1	1	2	1	1	1	8
Chen et al., 2021	1	1	0	2	1	1	1	7
Chun et al., 2016	1	1	0	2	1	1	1	7
Cwik & Singh 2021	1	1	0	2	1	1	1	7
Cwik & Singh 2022	1	1	0	2	1	1	1	7
Davis et al., 2019	1	1	0	1	0	1	1	5
Dueñas & Gloria 2020	1	0	1	2	1	1	1	7
Feser et al., 2024	1	0	0	2	1	1	1	6
Fink et al., 2020	1	1	0	2	1	1	1	7
Fong et al., 2023	1	1	0	2	1	1	1	7
Fong et al., 2024	1	1	0	2	1	1	1	7
Freeman 2007	1	0	0	2	0	1	1	5
Gan et al., 2024	1	1	0	2	1	1	1	7
Gaudier-Diaz et al., 2019	1	1	0	2	1	1	1	7
Gopalan & Brady 2020	1	1	0	1	1	1	1	6
Gulsen 2024	1	1	0	2	1	1	1	7
Hafer et al., 2021	1	1	0	2	1	1	1	7
Han et al., 2017	1	1	0	2	0	1	1	6
Han et al., 2022	1	1	0	2	1	1	1	7
Hartley 2011	1	1	0	2	1	1	1	7
Hausmann et al., 2009	1	0	1	2	1	1	1	7
Hilts et al., 2018	1	0	0	2	1	1	1	6
Jackson et al., 2023	1	1	0	1	1	1	1	6
Kahalon et al., 2023	1	0	0	1	1	1	1	5
Kelly et al., 2024	1	1	0	2	0	1	1	6
Kennedy & Tuckman 2013	1	1	0	2	1	1	1	7
Knekta et al., 2020	1	1	0	1	0	1	1	5
Krause-Levy et al., 2021	1	1	0	0	0	1	1	4
Lewis & Hodges 2015	1	1	0	1	0	1	1	5
Lewis et al., 2017	1	1	1	2	0	1	1	7
Li & Singh 2023	1	1	0	2	1	1	1	7
Liang et al., 2022	1	1	1	2	1	1	1	8
Luo & Zhou 2022	1	0	0	2	1	1	1	6
Lytle & Shin 2020	1	1	1	2	1	1	1	8
Maluenda-Albornoz et al., 2022	1	1	0	2	1	1	1	7
Marksteiner et al., 2019	1	0	0	2	1	1	1	6
Matheka 2024	1	1	0	2	0	1	1	6
McClain et al., 2021	1	1	0	2	1	1	1	7

Mcdermott et al., 2020	1	1	1	2	1	1	1	8
McGee & Kruger 2022	1	0	1	2	1	1	1	7
Menkor et al., 2021	1	1	0	2	0	1	1	6
Mtshweni 2024	1	0	0	2	1	1	1	6
Mtshweni 2024a	1	1	0	2	1	1	1	7
Okolie et al., 2021	1	1	0	2	0	1	1	6
Pedler et al., 2021	1	1	0	1	0	1	1	5
Pittman & Richmond 2007	1	0	0	2	1	1	1	6
Rattan et al., 2018	1	0	0	2	0	1	1	5
Reid et al., 2019	1	1	1	2	1	1	1	8
Reid et al., 2020	1	1	1	2	1	1	1	8
Saroughi & Kitsantas 2021	1	1	0	2	1	1	1	7
Schar et al., 2017	1	0	0	1	1	1	1	5
Seyranian et al., 2018	1	0	0	2	1	1	1	6
Shook & Clay 2012	1	0	0	1	0	1	1	4
Smith et al., 2012	1	0	0	1	0	1	1	4
Sotardi et al., 2022	1	1	1	2	1	1	1	8
Strayhorn 2020	1	0	0	1	1	1	1	5
Suhlmann et al., 2018	1	1	0	2	0	1	1	6
Swanson & Martorell 2021	1	1	0	2	1	1	1	7
Takimoto et al., 2021	1	0	0	2	1	1	1	6
Tao et al., 2008	1	0	0	2	1	1	1	6
Thomas et al., 2014	1	0	0	2	1	1	1	6
Townley et al., 2013	1	0	0	1	1	1	1	5
van Herpen et al., 2020	1	0	0	2	0	1	1	5
van Lamoen et al., 2024	1	1	1	2	1	1	1	8
Vayre & Vonthron 2018	1	1	0	2	1	1	1	7
Vazquez et al., 2024	1	1	0	2	1	1	1	7
Veldman et al., 2023	1	1	0	2	0	1	1	6
Verdin 2021	1	0	0	2	1	1	1	6
Wells & Horn 2015	1	0	0	2	0	1	1	5
Wilcox & Nordstokke 2019	1	0	0	2	0	1	1	5
Wilson et al., 2018	1	1	0	2	0	1	1	6
Wilson et al., 2020	1	1	0	2	1	1	1	7
Witherspoon & Schunn 2022	1	1	0	2	1	1	1	7
Wolf et al., 2021	1	1	0	2	1	1	1	7
Yust et al., 2021	1	1	1	2	1	1	1	8
Zumbrunn et al., 2014	1	0	0	2	1	1	1	6

Note. 1, Representativeness of the sample; 2, Sample size; 3, Non-respondents; 4, Ascertainment of the exposure; 5, Comparability; 6, Assessment of the outcome; 7, Statistical test.

Supplementary Material 3

Forest Plots

Study	ZCOR	SE(ZCOR)	COR	95%-CI	Weight
Au et al., 2023	-0.165	0.085	-0.16	[-0.32; 0.00]	1.8%
Banchefsky et al., 2019	0.618	0.044	0.55	[0.49; 0.61]	2.2%
Buskirk-Cohen & Plants, 2019	0.192	0.156	0.19	[-0.11; 0.46]	1.2%
Carey et al., 2022	0.192	0.049	0.19	[0.10; 0.28]	2.2%
Chen et al., 2021	0.224	0.042	0.22	[0.14; 0.30]	2.2%
Chun et al., 2016	-0.100	0.059	-0.10	[-0.21; 0.02]	2.1%
Cwik & Singh, 2021	0.266	0.034	0.26	[0.20; 0.32]	2.3%
Cwik & Singh, 2022	0.343	0.035	0.33	[0.27; 0.39]	2.3%
Davis et al., 2019	0.060	0.035	0.06	[-0.01; 0.13]	2.3%
Fink et al., 2020	0.321	0.037	0.31	[0.24; 0.37]	2.3%
Fong et al., 2023	0.136	0.027	0.14	[0.08; 0.19]	2.3%
Fong et al., 2024	0.151	0.020	0.15	[0.11; 0.19]	2.4%
Freeman, 2007	0.100	0.065	0.10	[-0.03; 0.22]	2.0%
Gan et al., 2024	0.341	0.015	0.33	[0.30; 0.35]	2.4%
Gaudier-Diaz et al., 2019	-0.020	0.036	-0.02	[-0.09; 0.05]	2.3%
Gopalan & Brady, 2020	0.100	0.006	0.10	[0.09; 0.11]	2.4%
Hafer et al., 2021	-0.020	0.033	-0.02	[-0.08; 0.04]	2.3%
Han et al., 2017	0.266	0.027	0.26	[0.21; 0.31]	2.3%
Hartley, 2011	-0.060	0.041	-0.06	[-0.14; 0.02]	2.2%
Kahalon et al., 2023	0.141	0.107	0.14	[-0.07; 0.34]	1.6%
Kennedy & Tuckman, 2013	0.050	0.022	0.05	[0.01; 0.09]	2.3%
Lewis & Hodges, 2015	0.141	0.030	0.14	[0.08; 0.20]	2.3%
Li & Singh, 2023	0.412	0.043	0.39	[0.32; 0.46]	2.2%
Luo & Zhou, 2022	0.161	0.064	0.16	[0.04; 0.28]	2.0%
Marksteiner et al., 2019	-0.105	0.099	-0.10	[-0.29; 0.09]	1.7%
Pittman & Richmond , 2007	0.213	0.062	0.21	[0.09; 0.32]	2.0%
Rattan et al., 2018	0.460	0.123	0.43	[0.22; 0.61]	1.4%
Reid et al., 2019	0.213	0.017	0.21	[0.18; 0.24]	2.4%
Reid et al., 2020	0.151	0.051	0.15	[0.05; 0.25]	2.1%
Schar et al., 2017	0.234	0.112	0.23	[0.01; 0.42]	1.5%
Seyranian et al., 2018	0.100	0.080	0.10	[-0.06; 0.25]	1.9%
Shook & Clay, 2012	0.030	0.080	0.03	[-0.13; 0.18]	1.9%
Smith et al., 2012	0.460	0.083	0.43	[0.29; 0.55]	1.8%
Sotardi et al., 2022	0.040	0.033	0.04	[-0.02; 0.10]	2.3%
Swanson & Martorell , 2021	0.183	0.024	0.18	[0.14; 0.23]	2.3%
Tao et al., 2008	0.234	0.079	0.23	[0.08; 0.37]	1.9%
Townley et al., 2013	0.053	0.141	0.05	[-0.22; 0.32]	1.3%
van Herpen et al., 2020	-0.020	0.065	-0.02	[-0.15; 0.11]	2.0%
van Lamoen et al., 2024	0.020	0.022	0.02	[-0.02; 0.06]	2.3%
Vayre & Vonthron, 2018	0.012	0.045	0.01	[-0.08; 0.10]	2.2%
Vazquez et al., 2024	0.192	0.021	0.19	[0.15; 0.23]	2.3%
Wells & Horn, 2015	-0.151	0.094	-0.15	[-0.32; 0.03]	1.7%
Wilson et al., 2018	0.161	0.041	0.16	[0.08; 0.24]	2.2%
Wilson et al., 2020	0.203	0.046	0.20	[0.11; 0.29]	2.2%
Witherspoon & Schunn , 2022	0.400	0.049	0.38	[0.29; 0.46]	2.2%
Wolf et al., 2021	0.018	0.033	0.02	[-0.05; 0.08]	2.3%
Yust et al., 2021	0.255	0.044	0.25	[0.17; 0.33]	2.2%
Zumbrunn et al., 2014	0.060	0.069	0.06	[-0.08; 0.19]	2.0%
**Random effects model**			**0.15**	**[0.11; 0.20]**	**100.0%**

Heterogeneity: $I^2 = 93.9\%$, $\tau^2 = 0.0229$, $p < 0.0001$

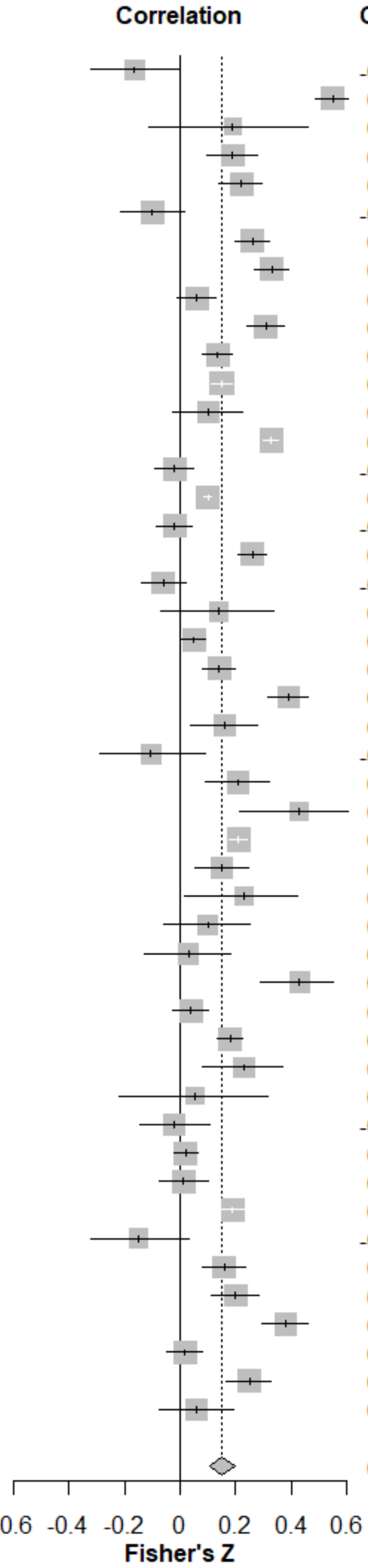

Fig S1. Academic Performance Forest Plots

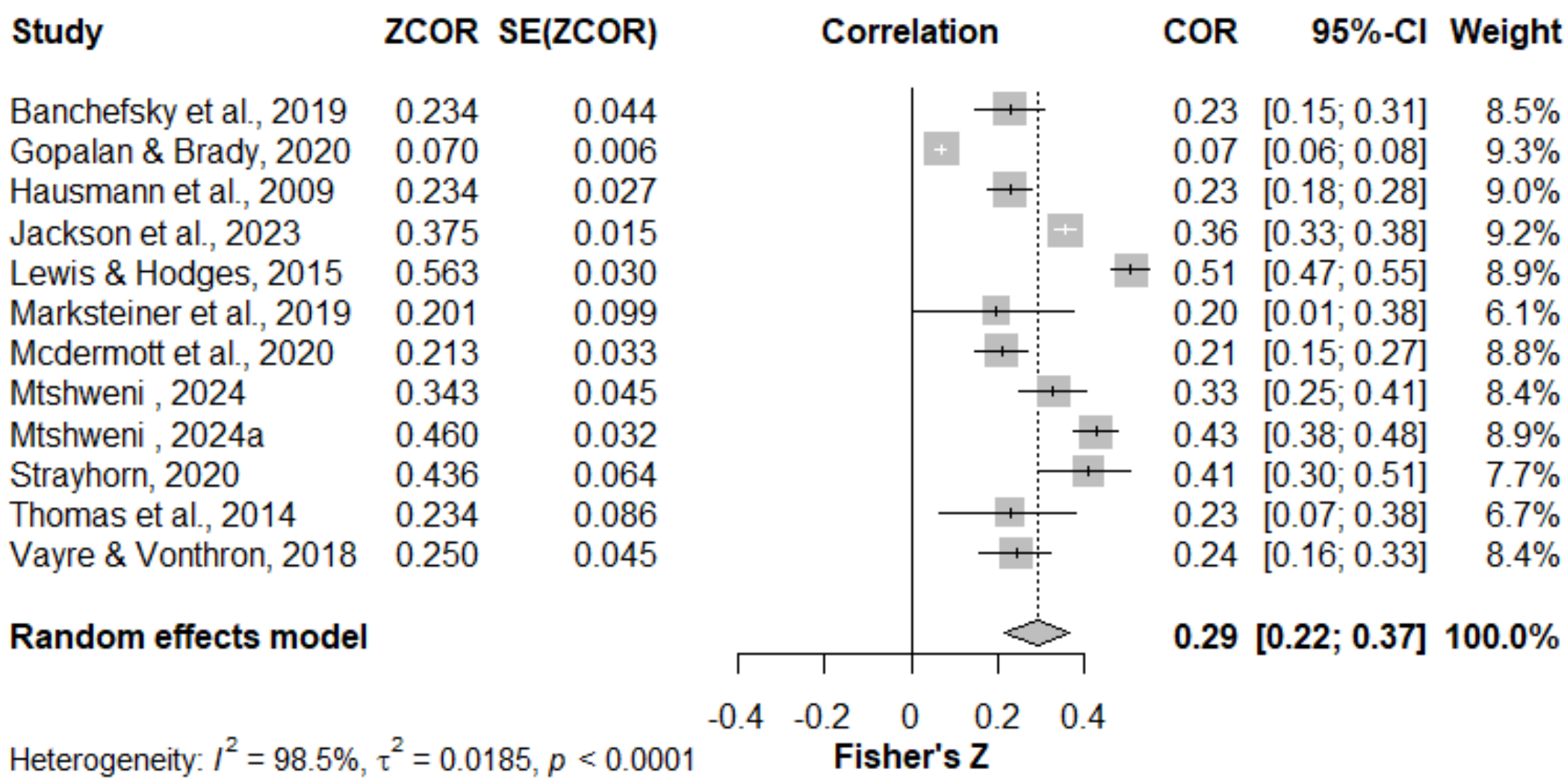

Fig S2. Academic Persistence Forest Plots

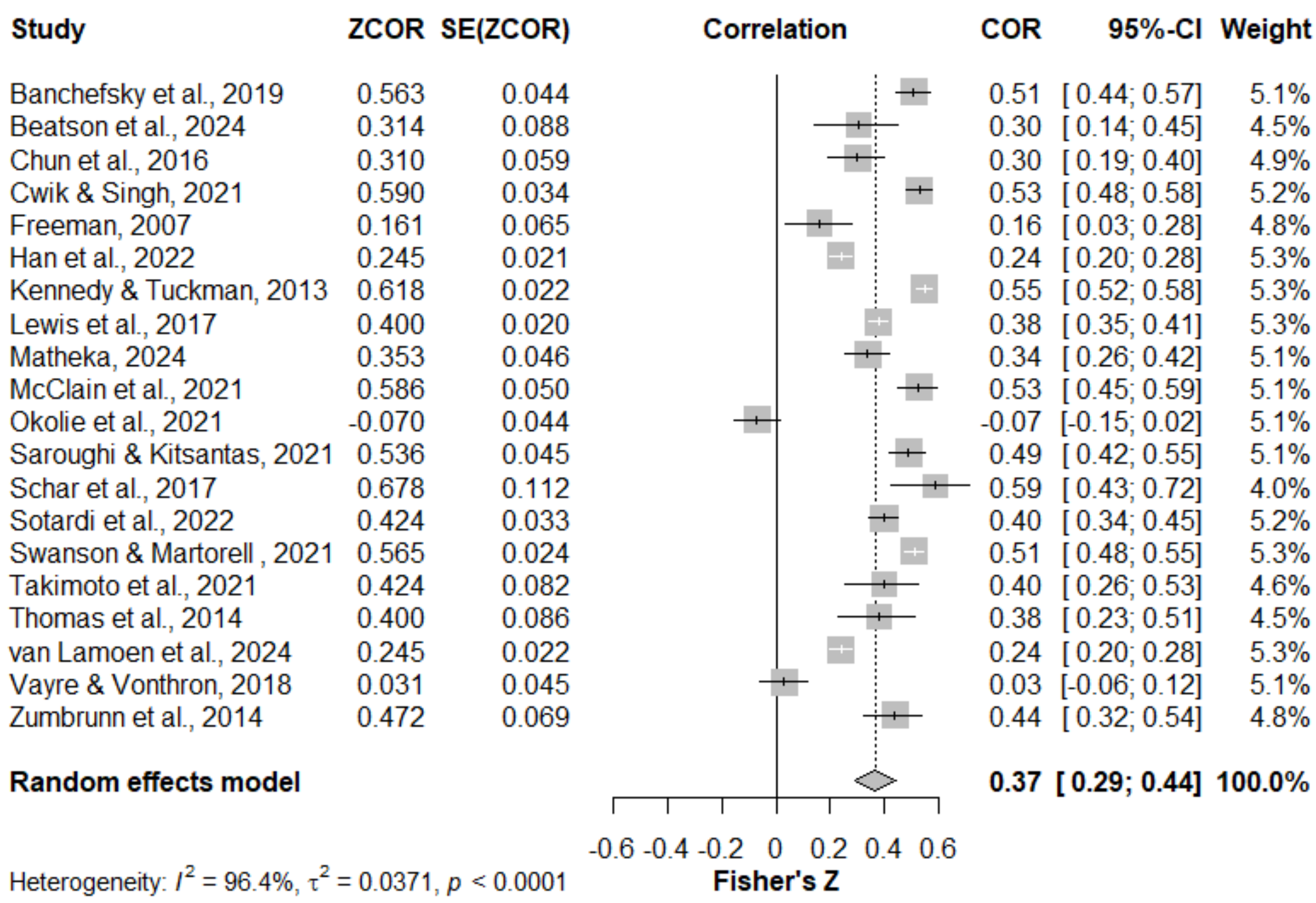

Fig S3. Academic Self-efficacy Forest Plots

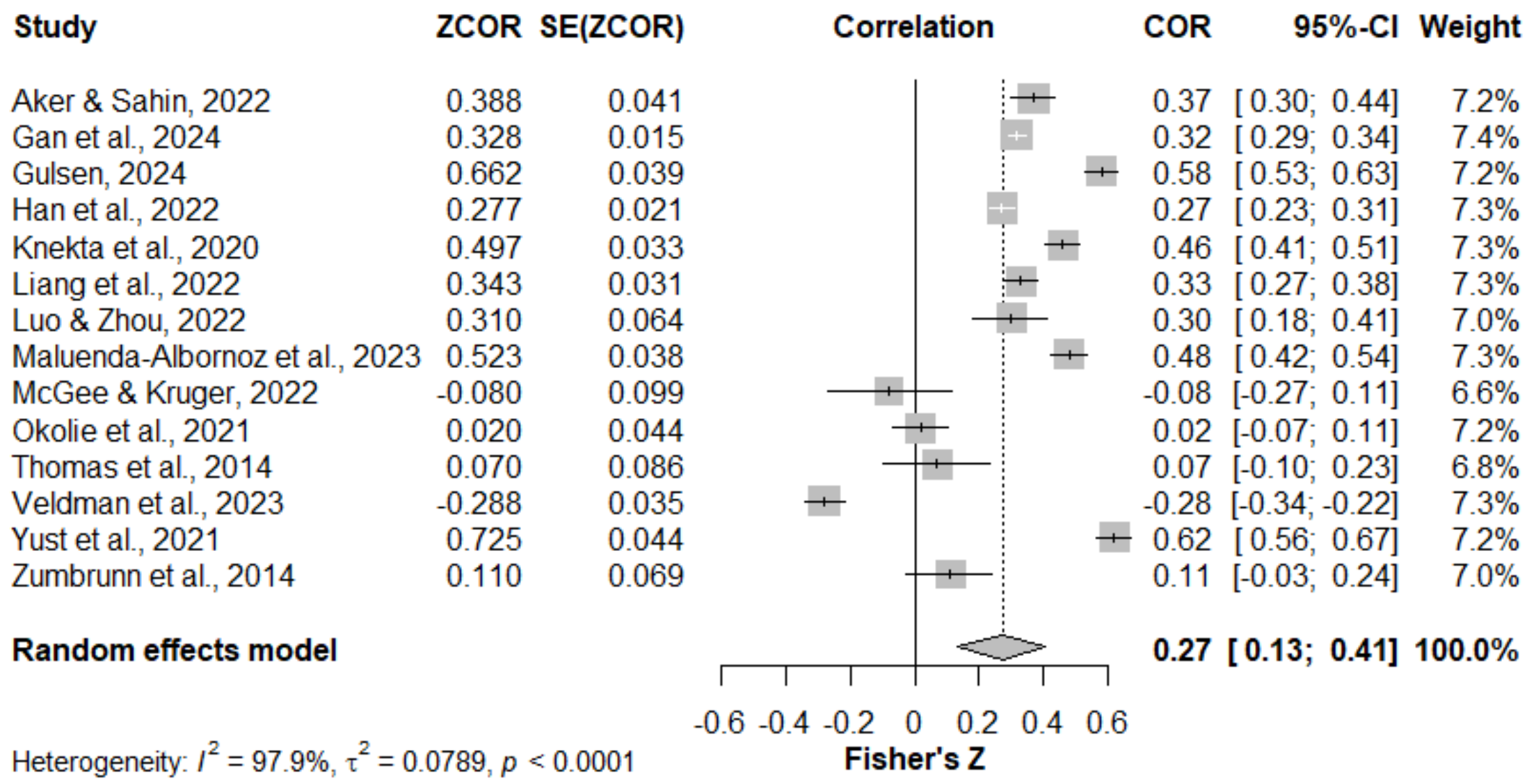

Fig S4. Academic Engagement Forest Plots

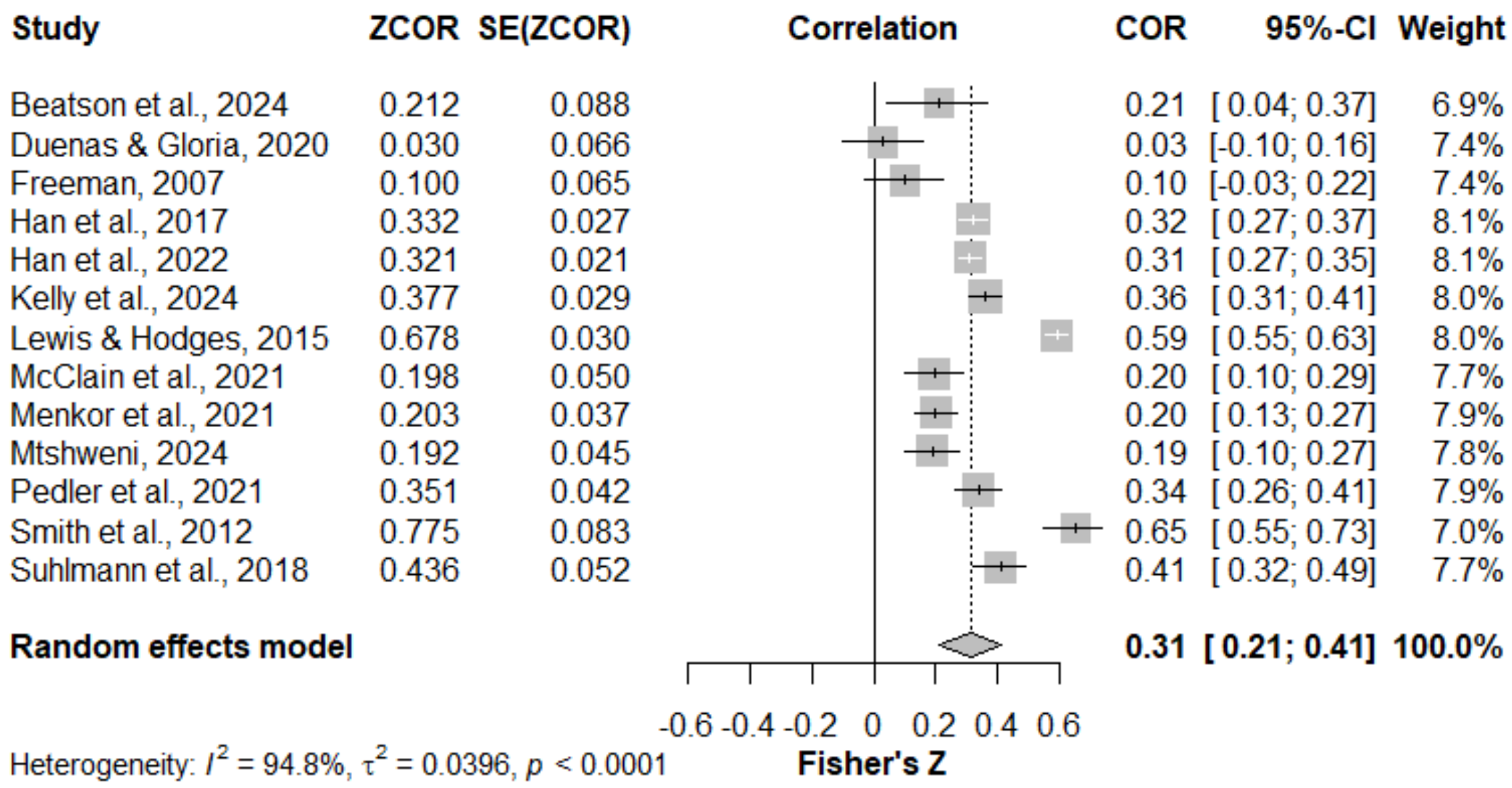

Fig S5. Academic Motivation Forest Plots

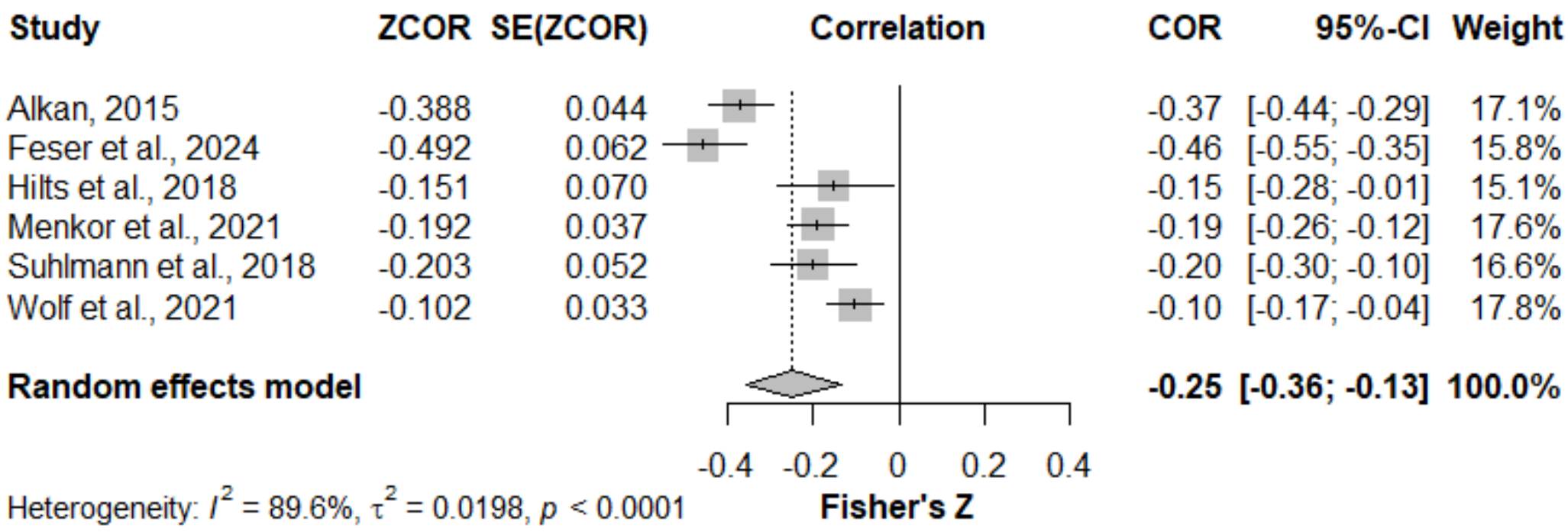

Fig S6. Dropout Intention Forest Plots

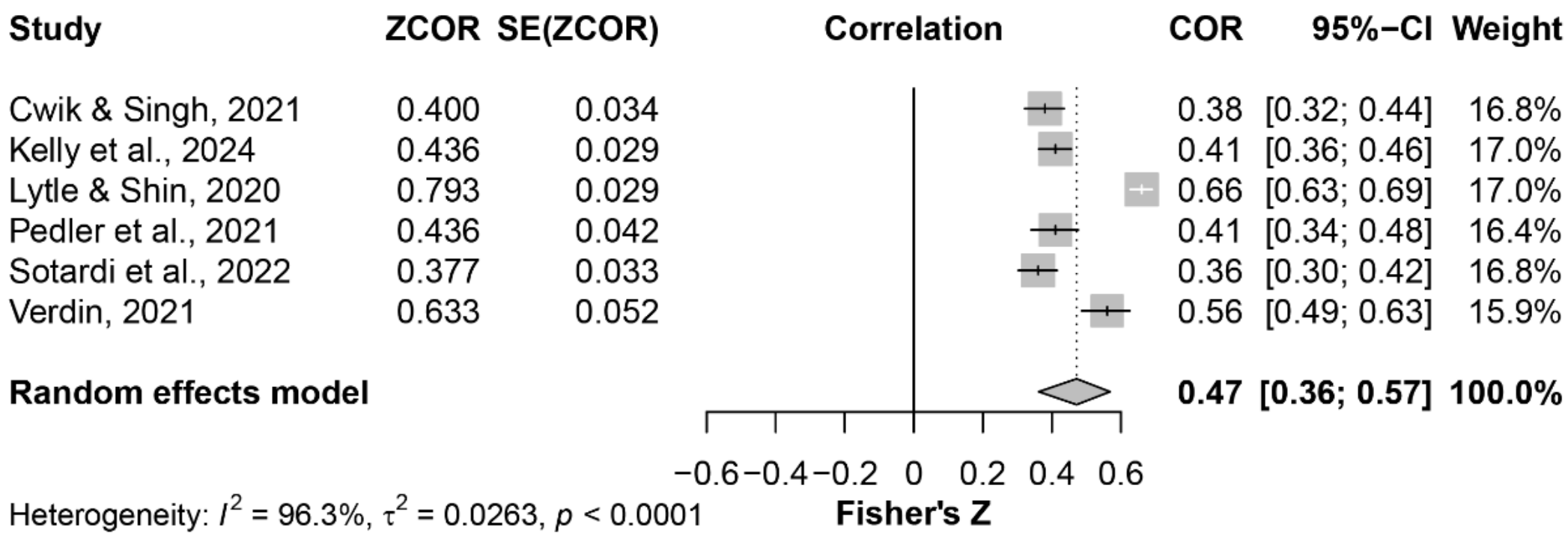

Fig S7. Academic Interest Forest Plots

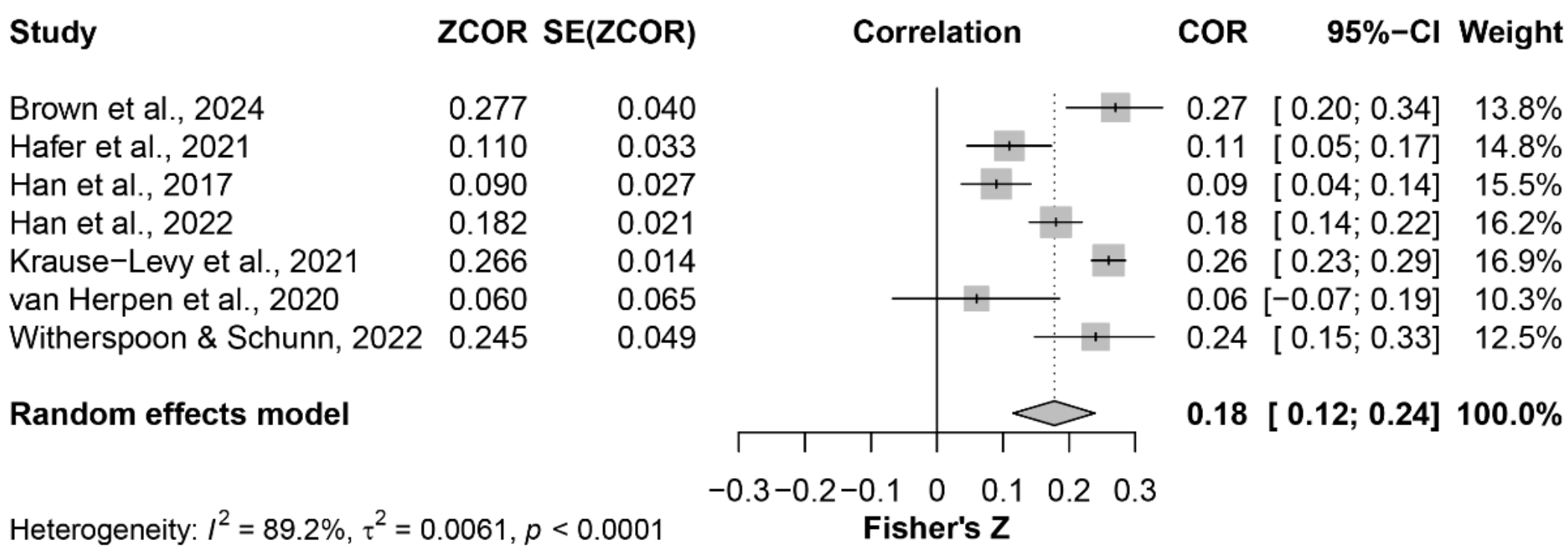

Fig S8. Student Retention Forest Plots

Supplementary Material 4

Funnel Plots

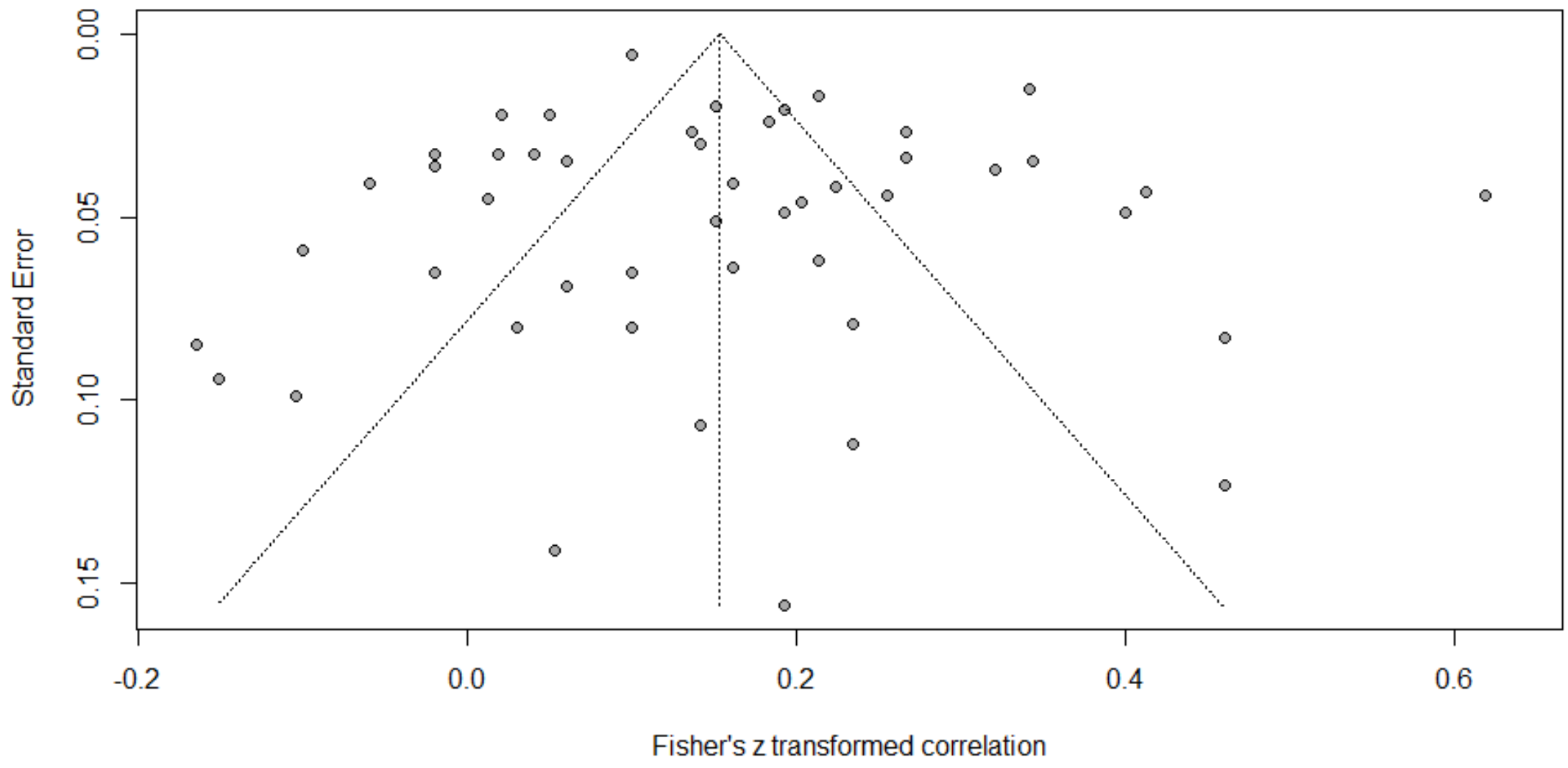

Fig S9. Academic Performance Funnel Plots

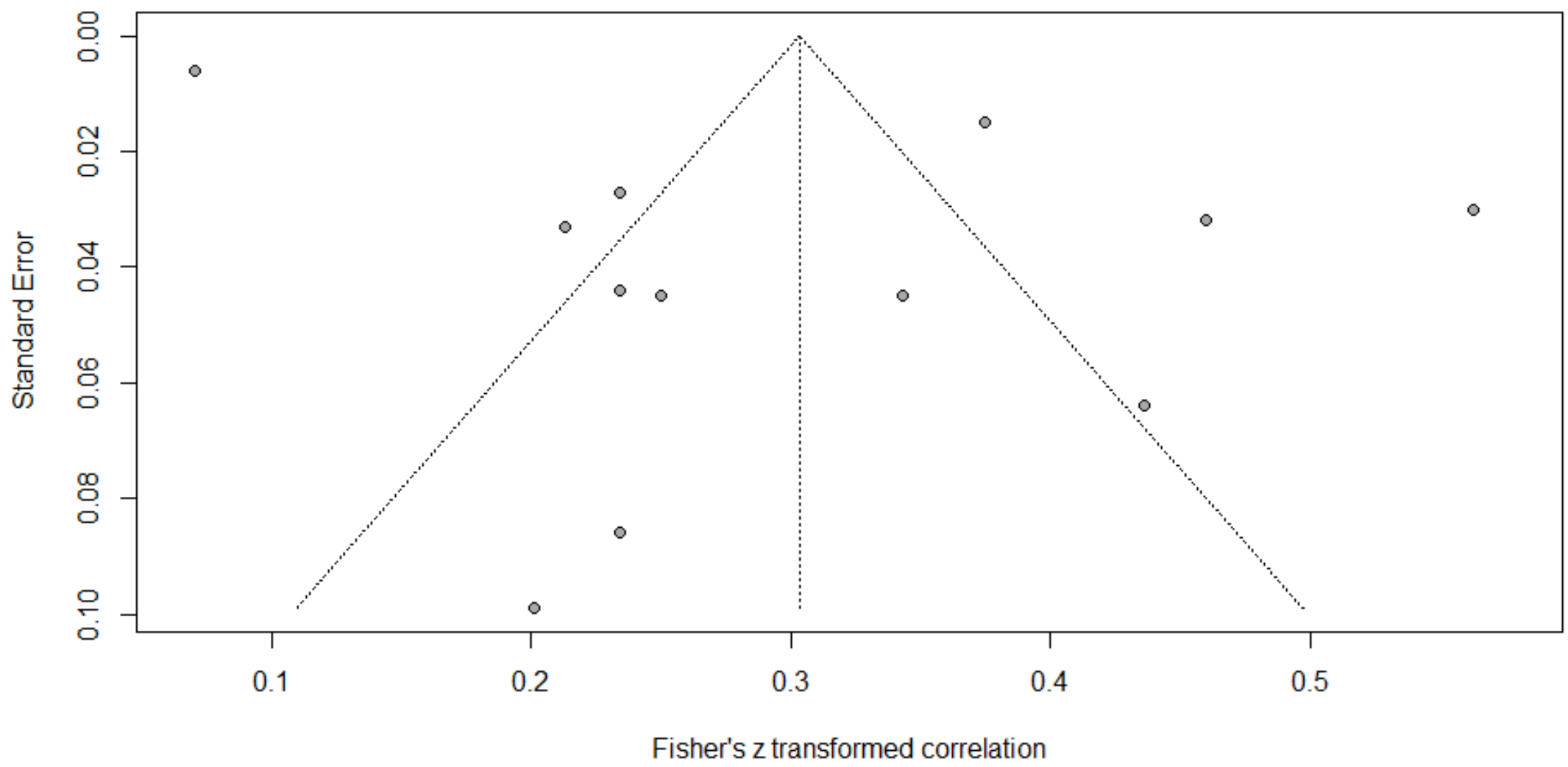

Fig S10. Academic Persistence Funnel Plots

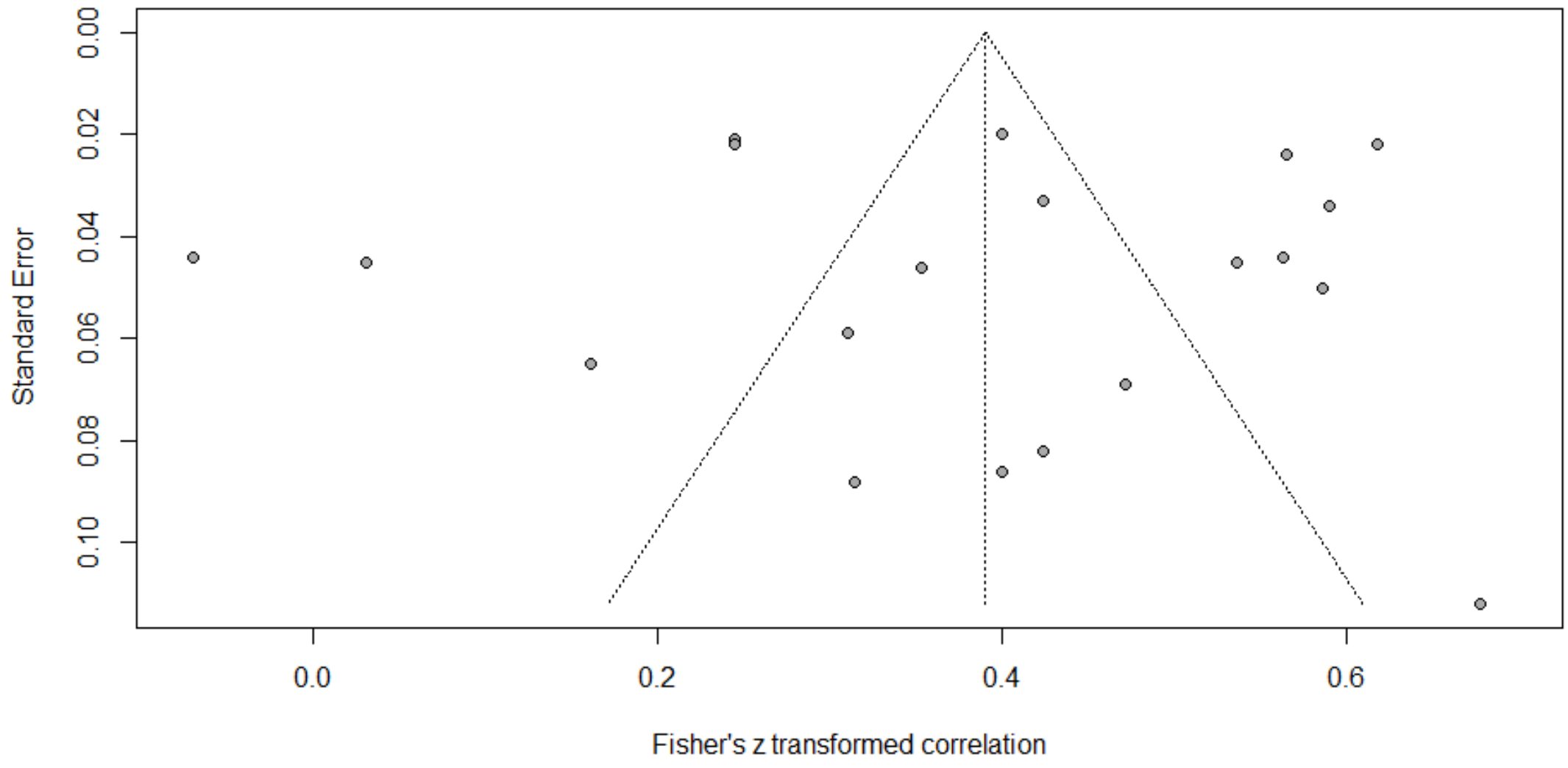

Fig 11. Academic Self-efficacy Funnel Plots

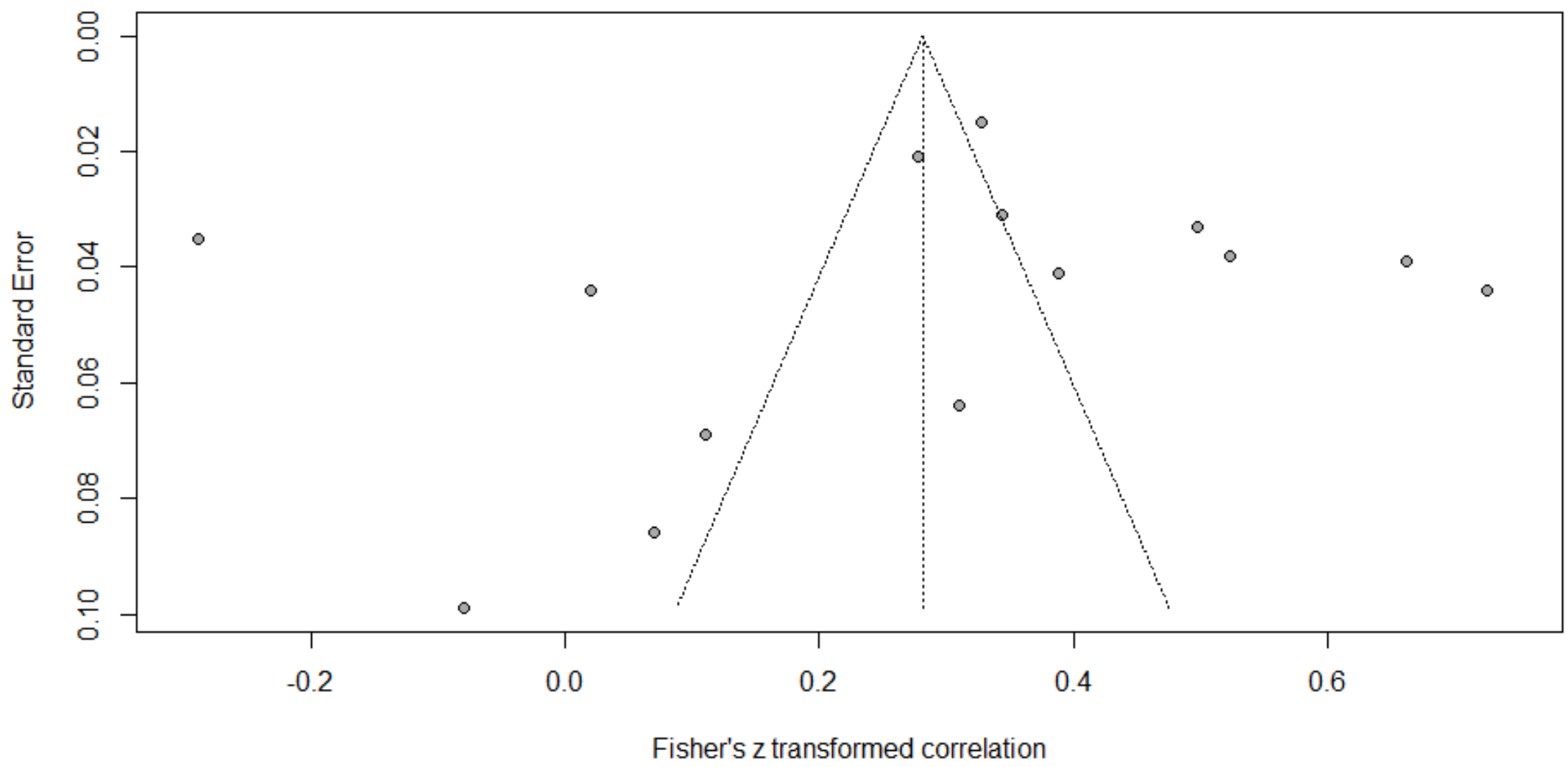

Fig 12. Academic Engagement Funnel Plots

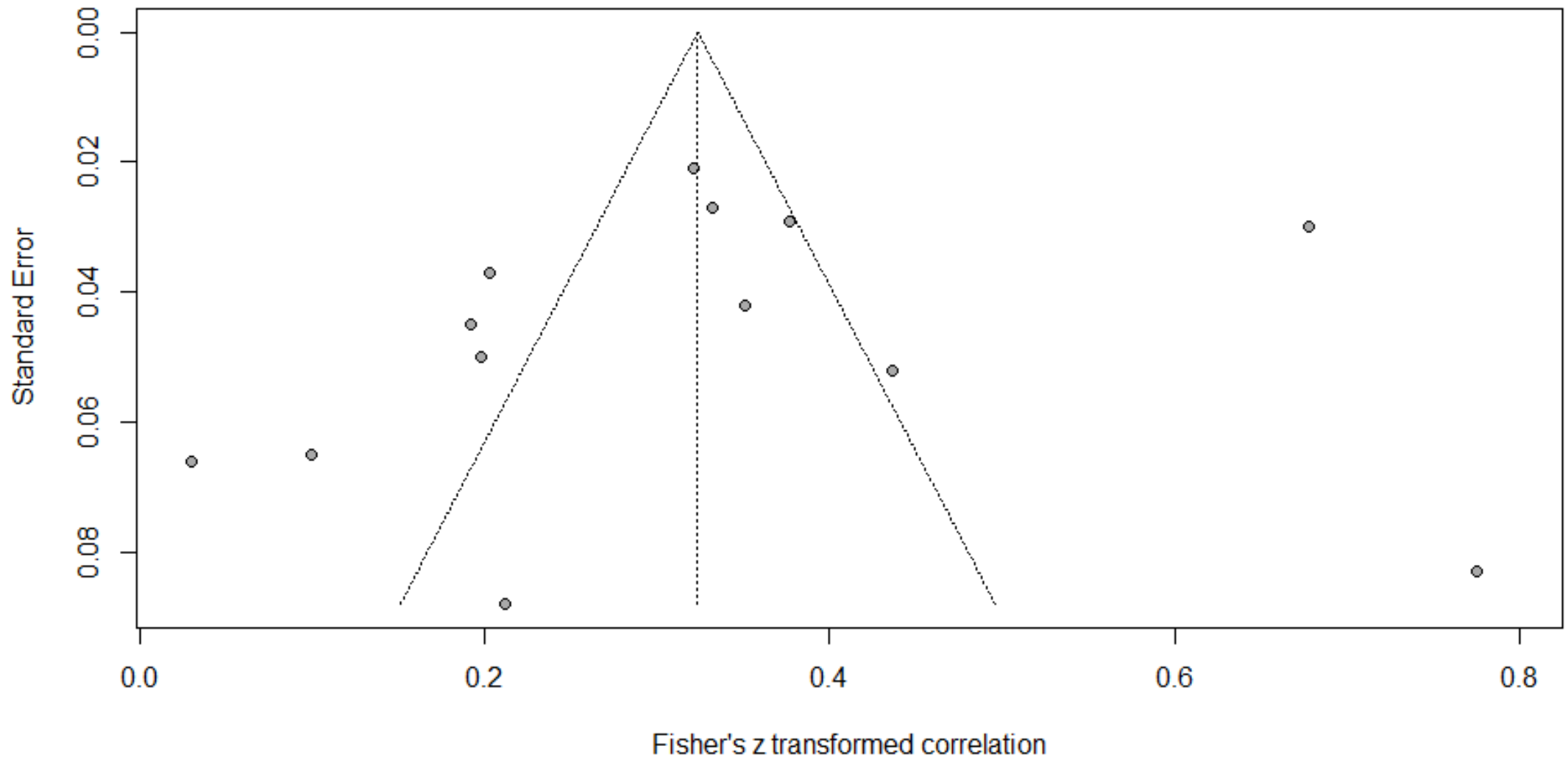

Fig 13. Academic Motivation Funnel Plots

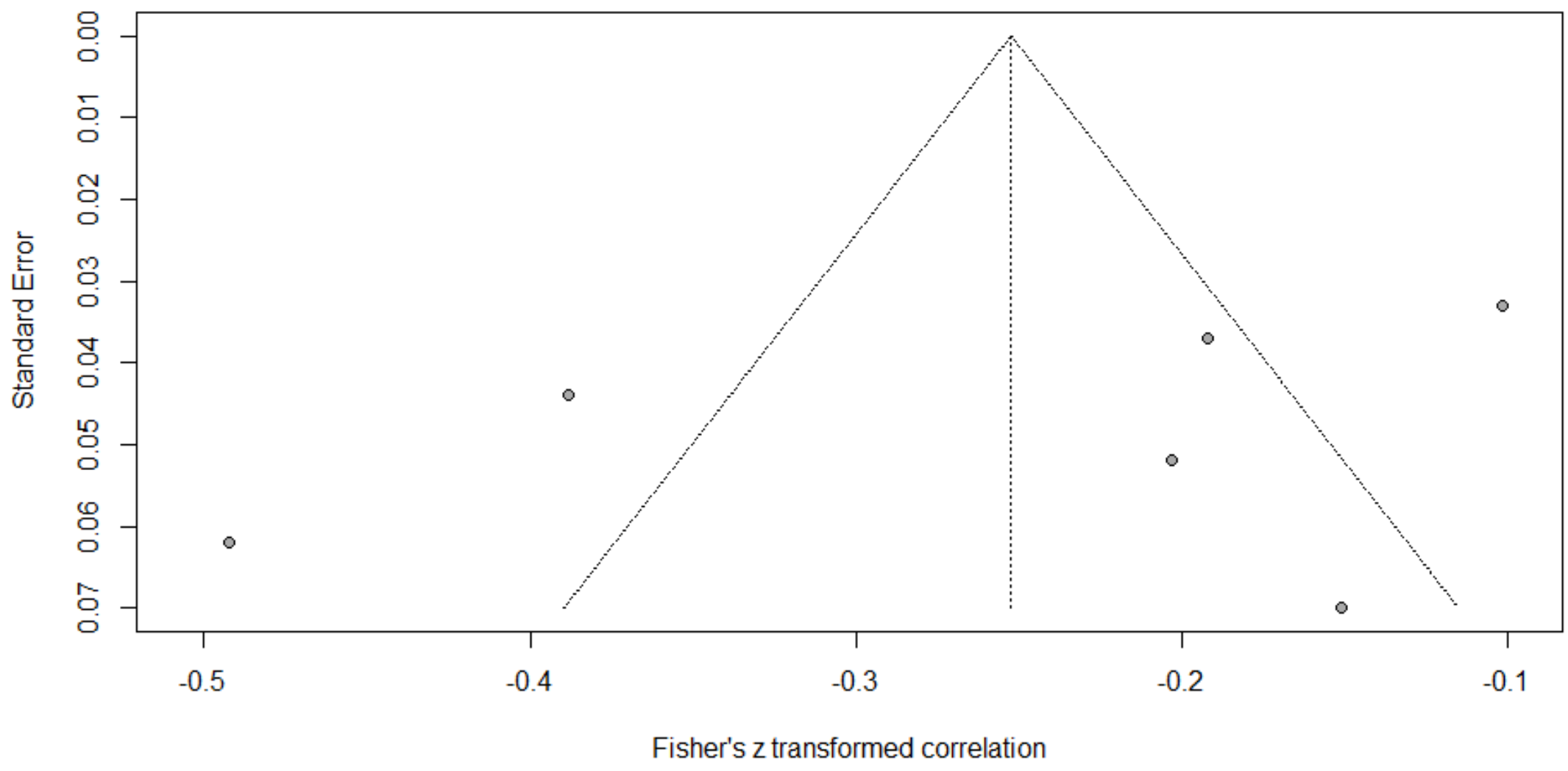

Fig S14. Dropout Intention Funnel Plots

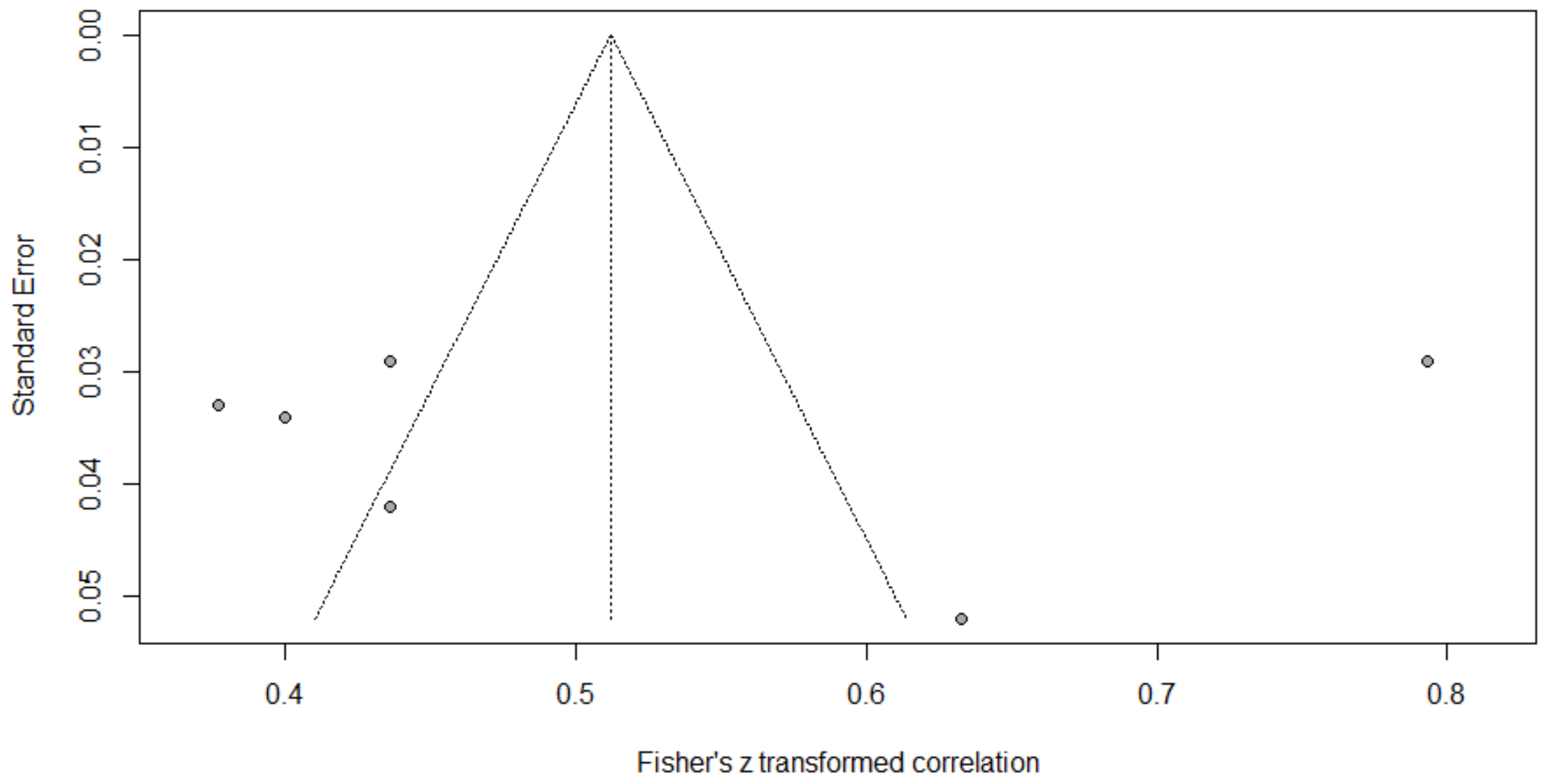

Fig S15. Academic Interest Funnel Plots

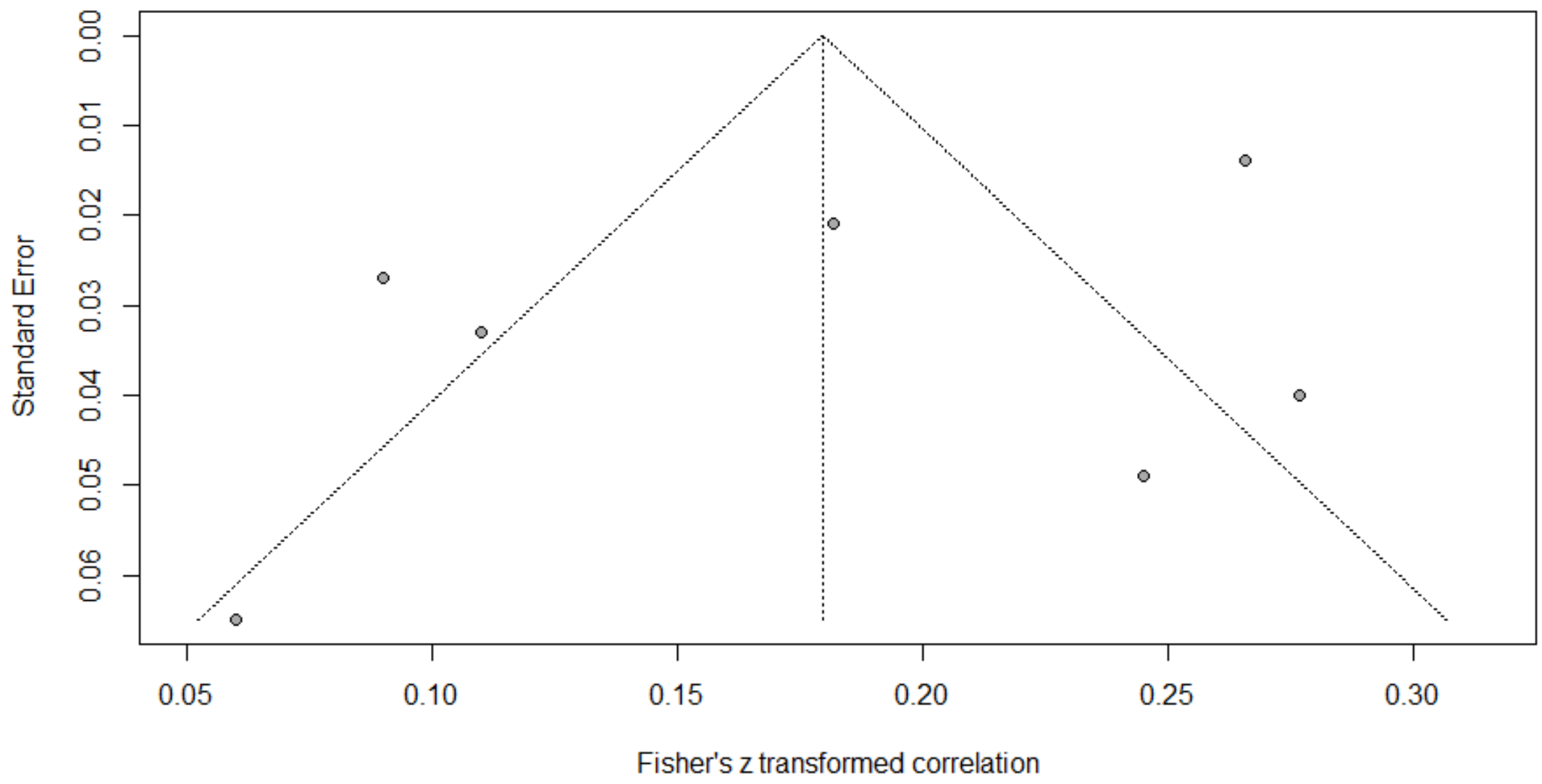

Fig S16. Student Retention Funnel Plots

Supplementary Material 5

Leave-one-out sensitivity analysis

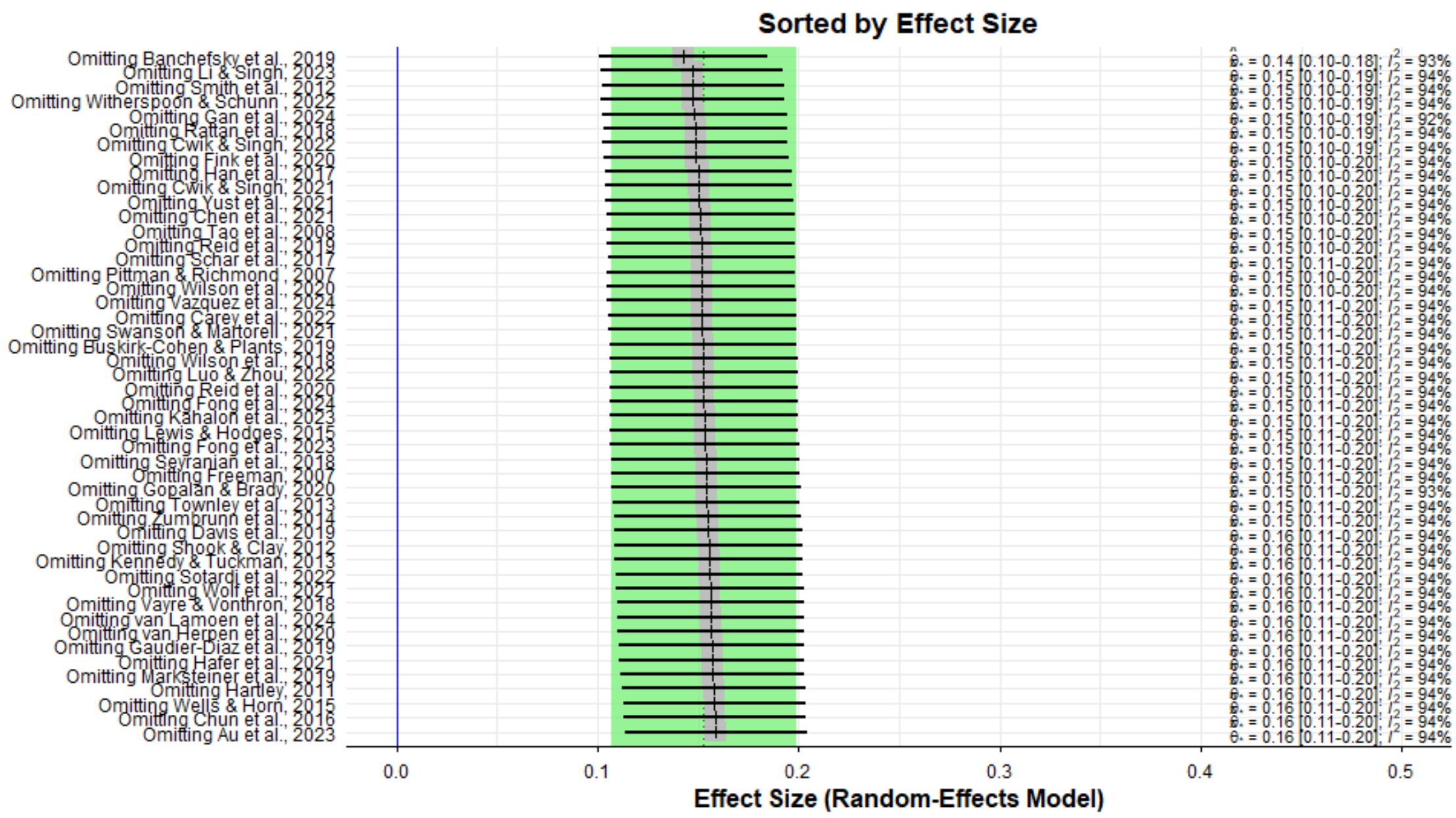

Fig S17. Academic Performance Sensitivity Analysis

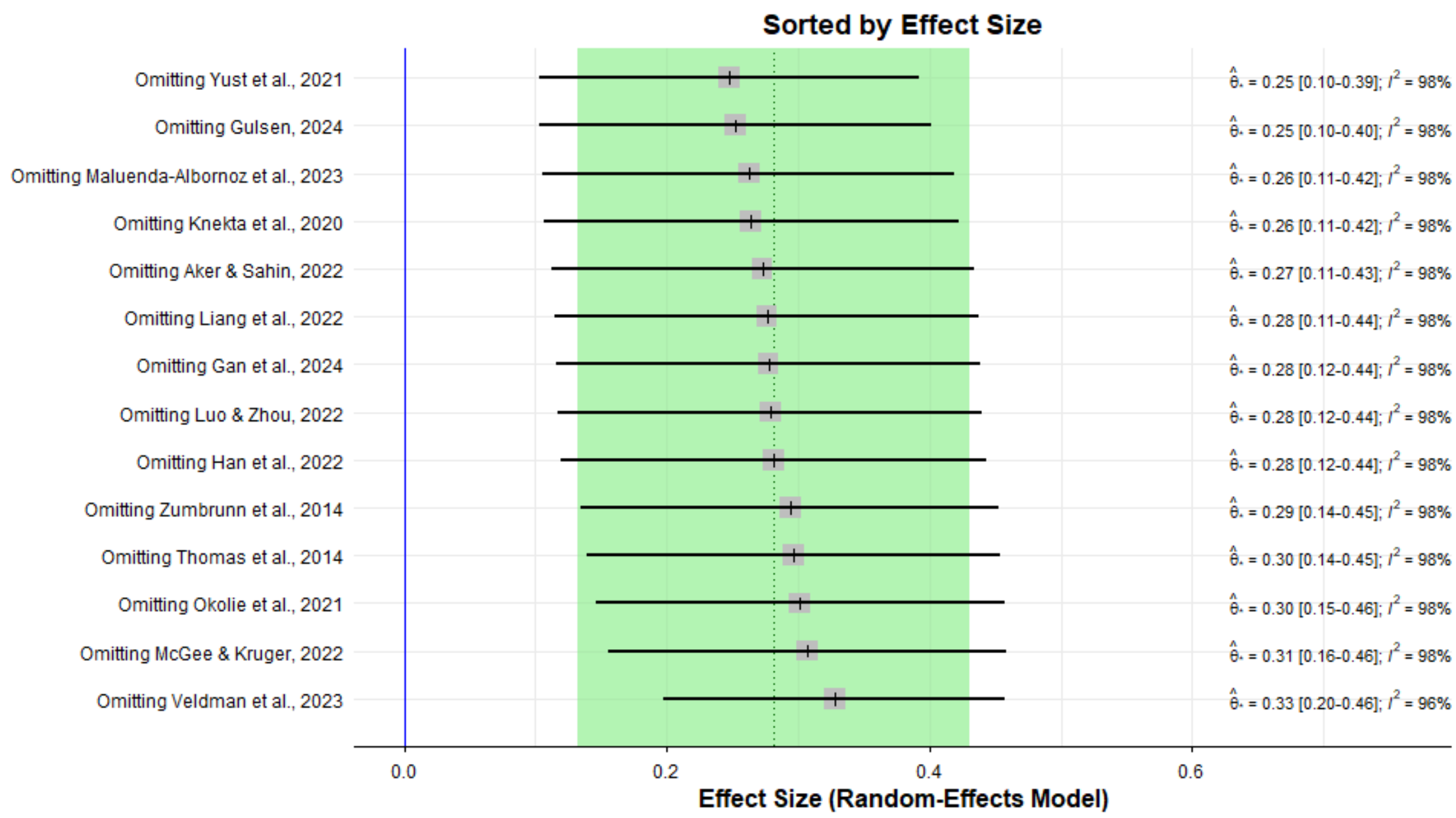

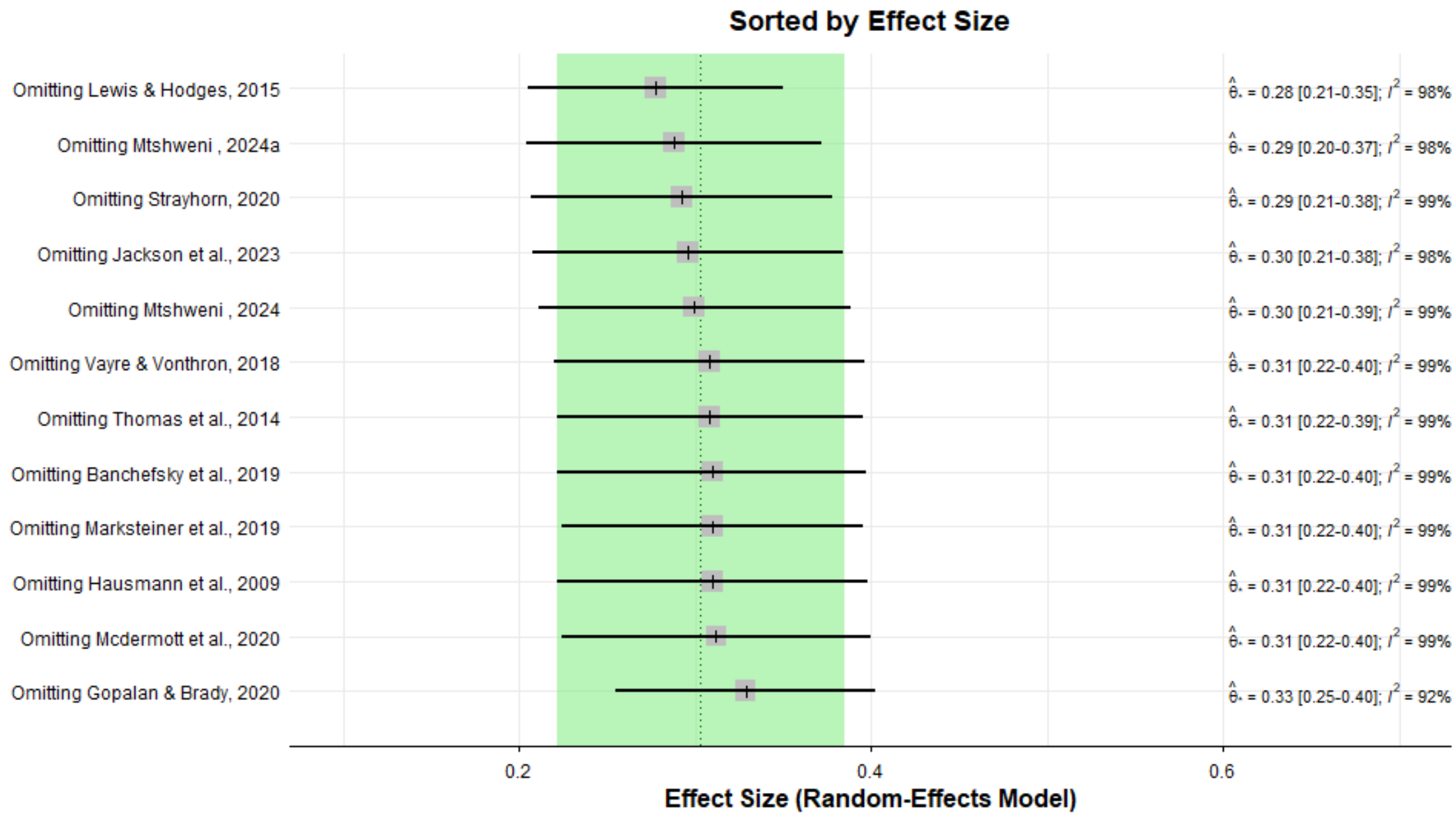

Fig S10. Academic Persistence Funnel Plots

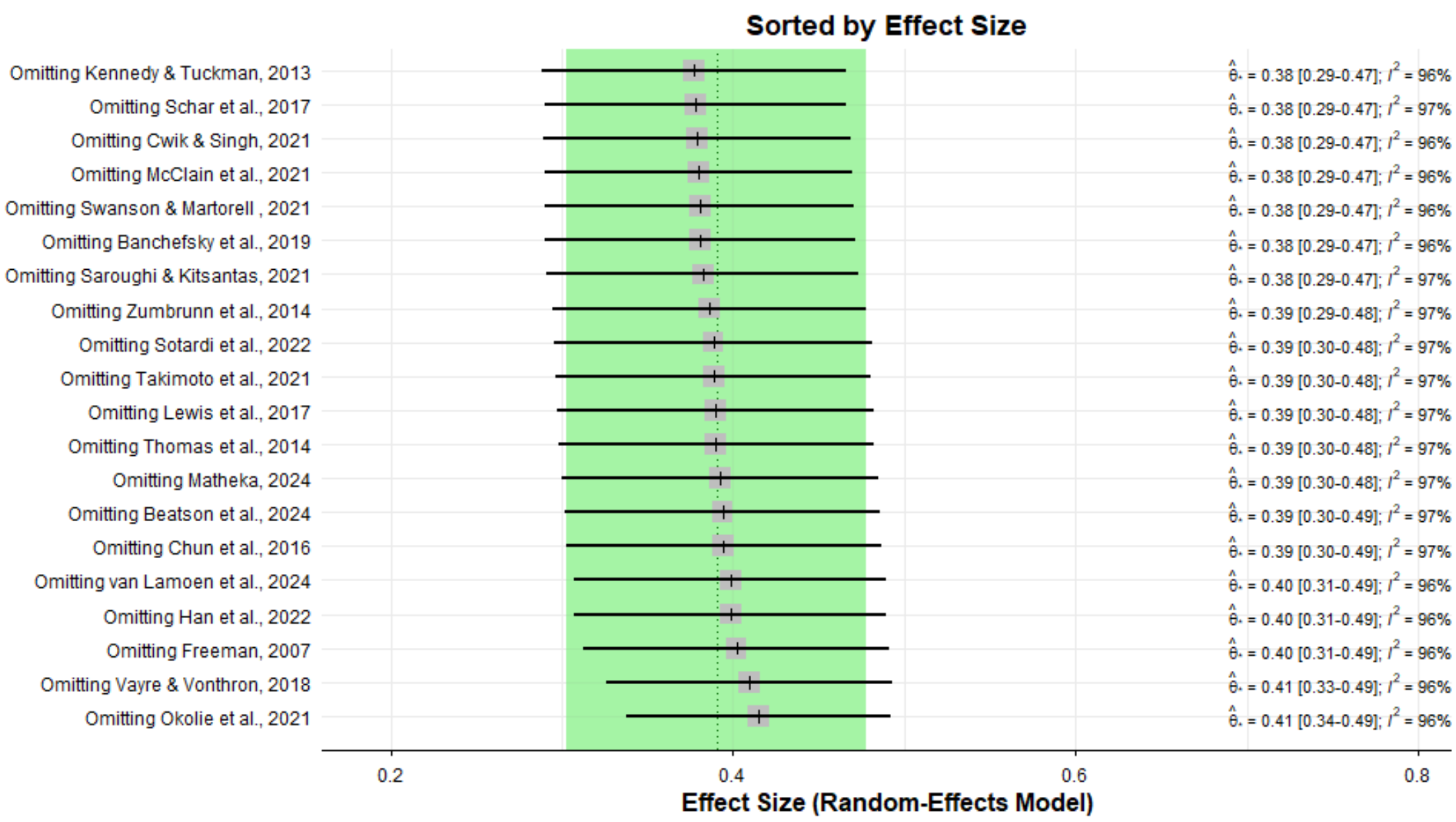

Fig 11. Academic Self-efficacy Funnel Plots

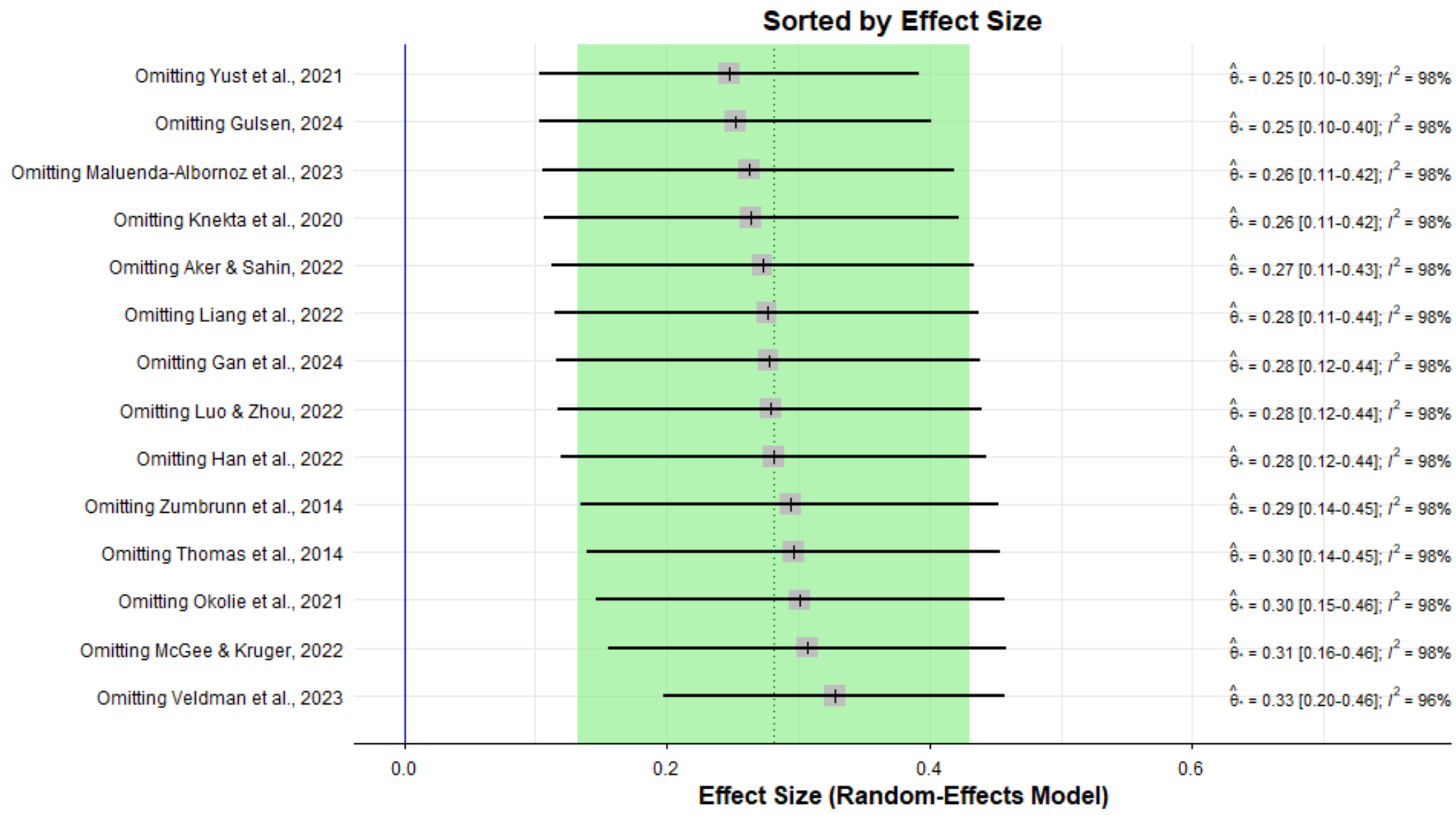

Fig 12. Academic Engagement Funnel Plots

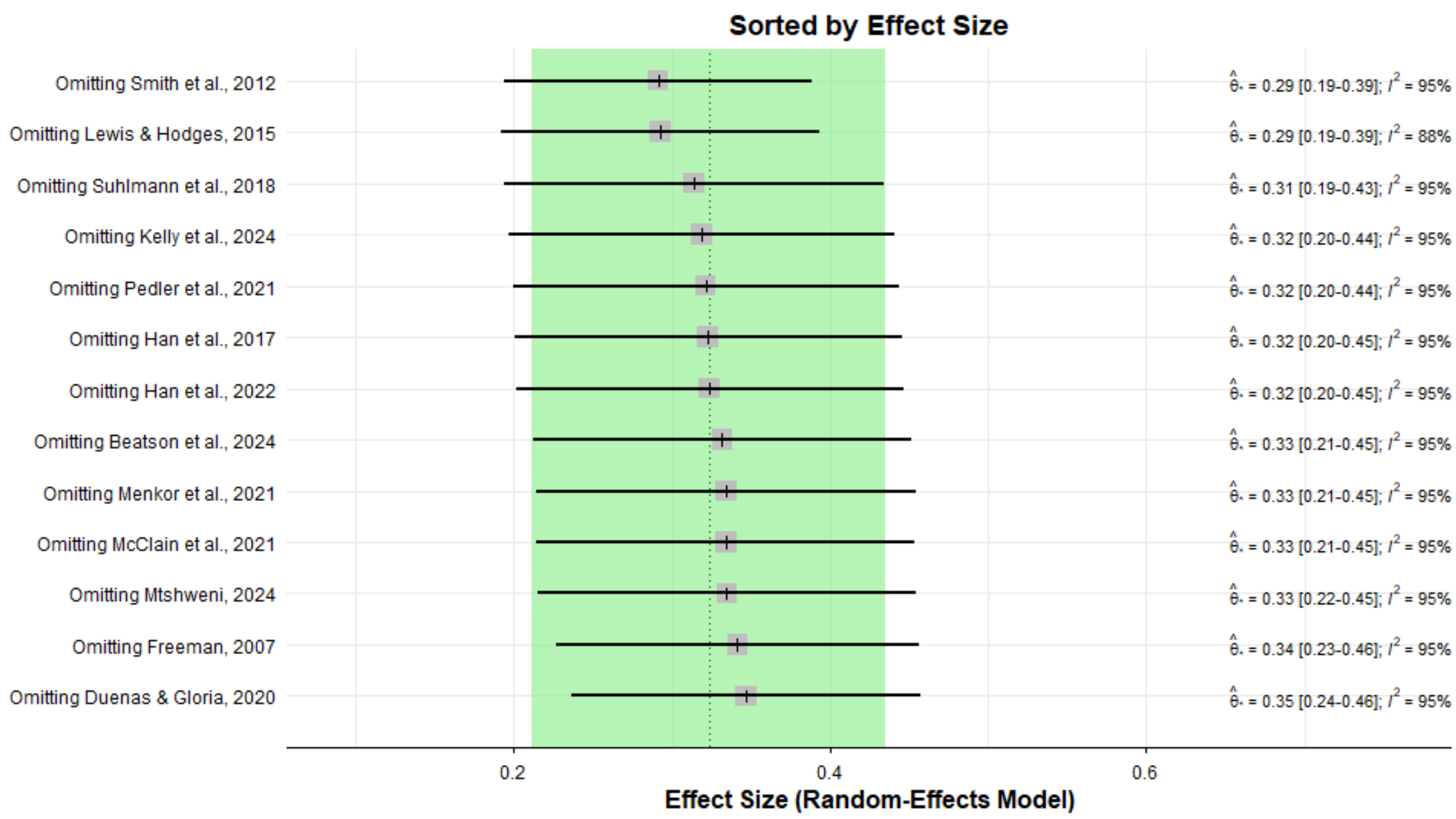

Fig 13. Academic Motivation Funnel Plots

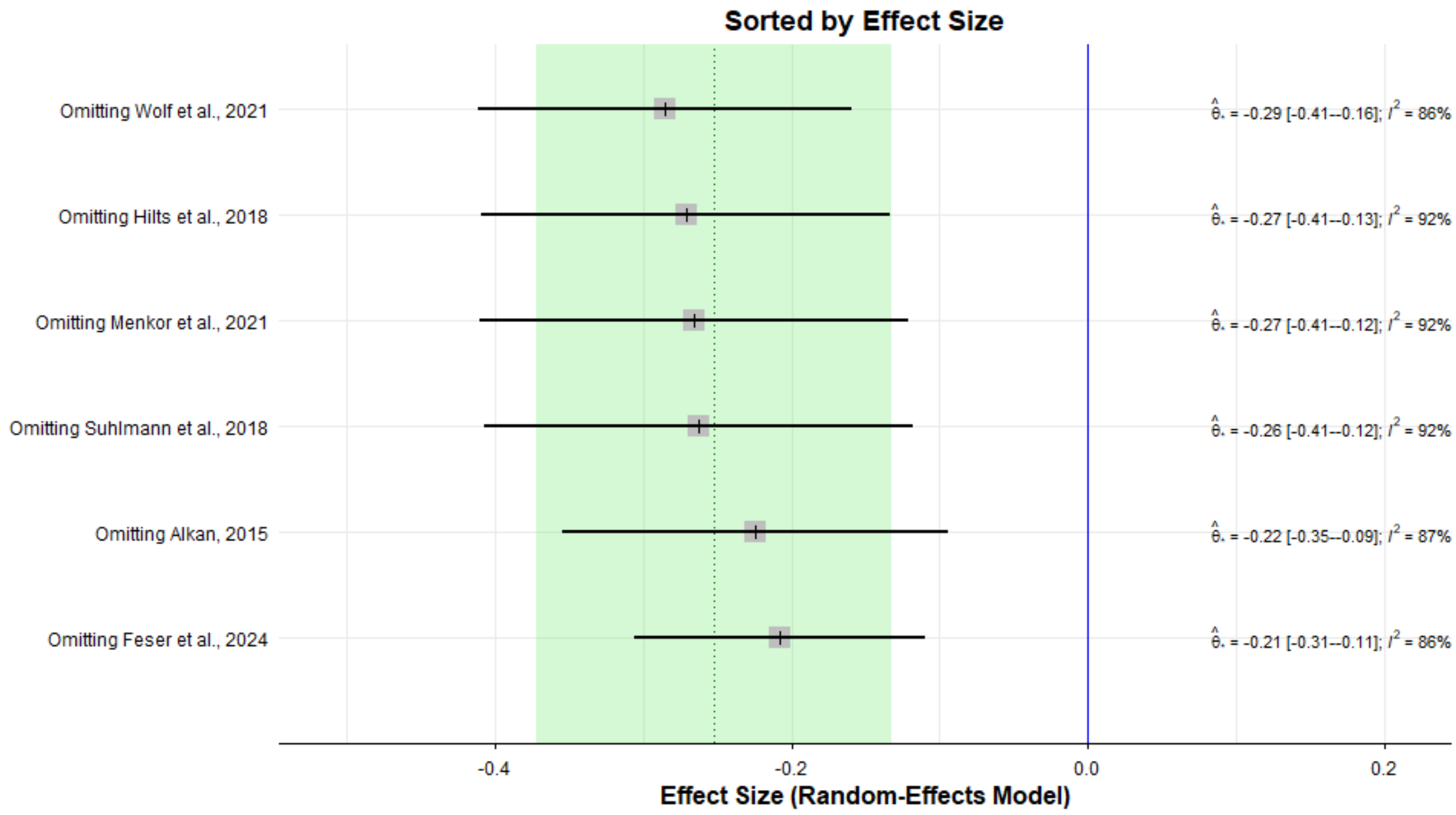

Fig S14. Dropout Intention Funnel Plots

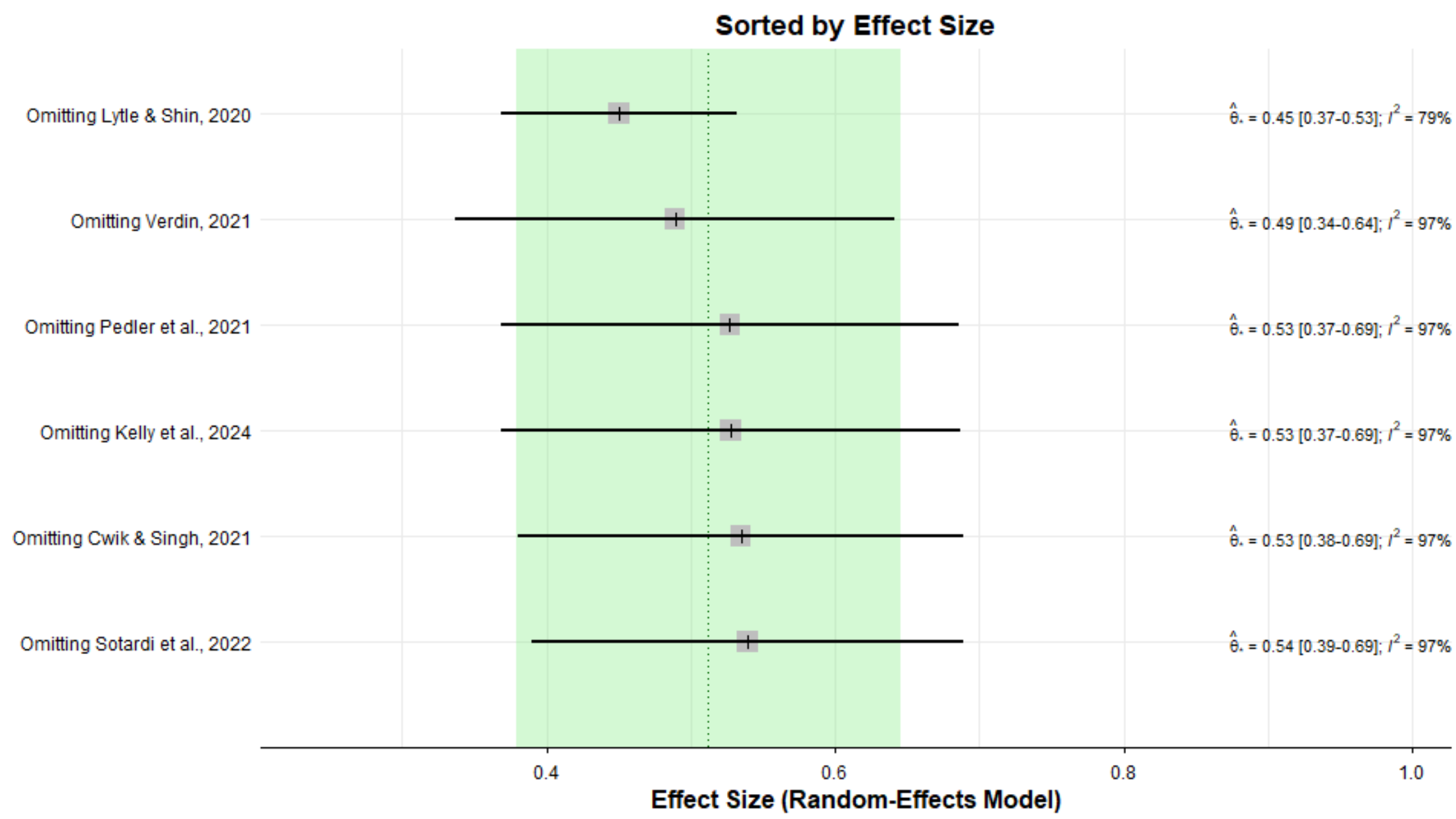

Fig S15. Academic Interest Funnel Plots

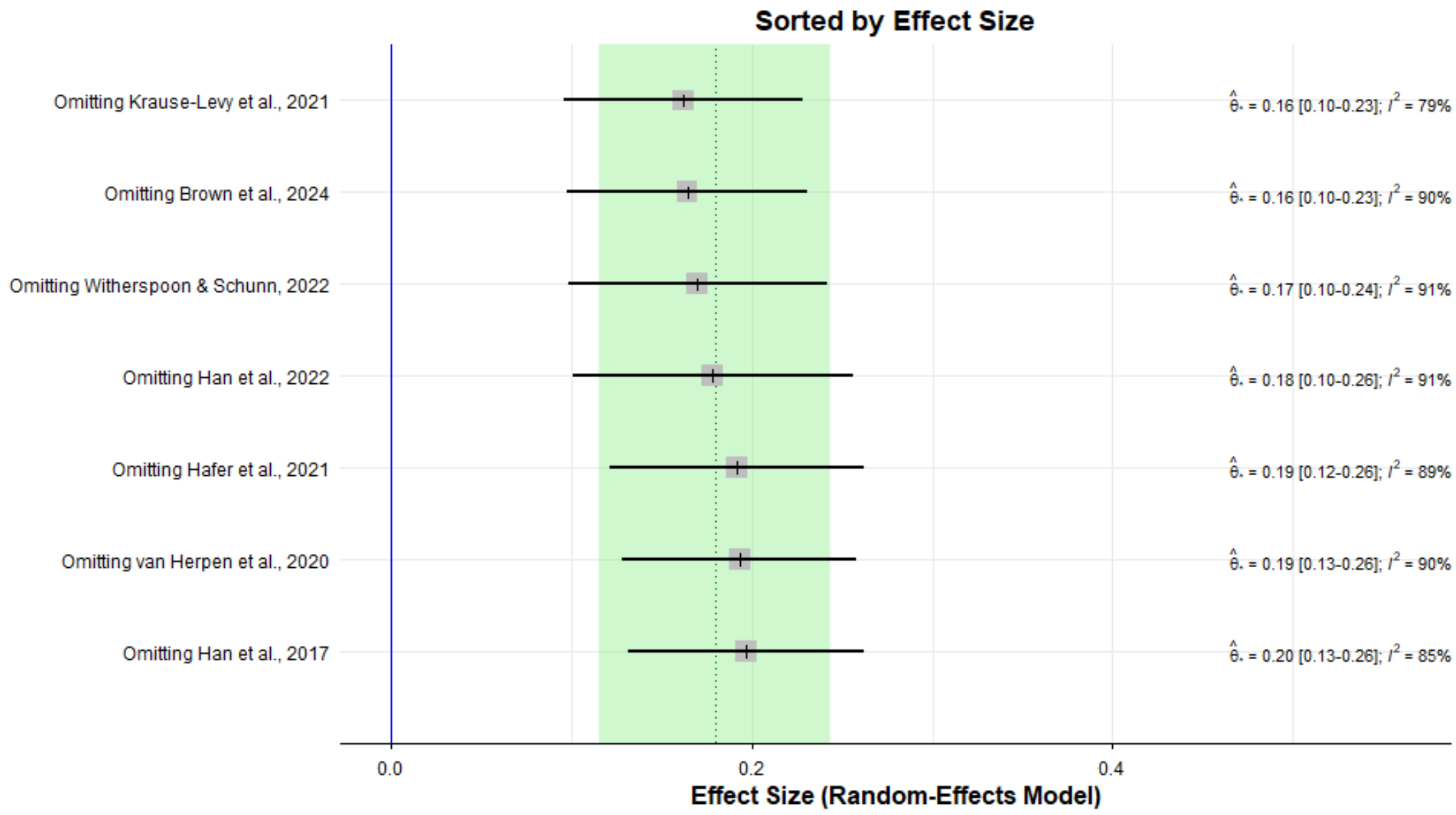

Fig S16. Student Retention Funnel Plots
